# Transdisciplinary training to address challenges in genomic epidemiology of infectious diseases

**DOI:** 10.3389/fpubh.2025.1713182

**Published:** 2025-12-17

**Authors:** Samuel L. Hong, Marie Stockman, Jorge Ricardo Nova Blanco, Casper Van Cleemput, Ambroise Ahouidi, Blas Armién, Ana Bento, Nena Bollen, César Conde Pereira, Carla Freitas, Marije Hofstra, Michael R. Jordan, Simone Kashima, Crhistinne Cavalheiro Maymone Gonçalves, Carlos Frederico Campelo de Albuquerque e Melo, Kanika Nahata, Elaine Cristina de Oliveira, Liz Parra, Carlos Saenz, Walban de Souza, Maja Stanojevic, Peter MacGarr Rabinowitz, Anne-Mieke Vandamme

**Affiliations:** 1 Laboratory of Evolutionary and Computational Virology, Department of Microbiology, Immunology and Transplantation, Rega Institute for Medical Research, KU Leuven, Leuven, Belgium; 2 Department of Microbiology, Immunology and Transplantation, Rega Institute for Medical Research, Clinical and Epidemiological Virology, Institute for the Future, KU Leuven, Leuven, Belgium; 3 Institut de Recherche en Santé, de Surveillance Epidemiologique et de Formations (IRESSEF), Dakar, Senegal; 4 Departamento de Investigación de Enfermedades Emergentes y Zoonóticas - DIEEZ-ICGES, Instituto Conmemorativo Gorgas de Estudios de la Salud, Ciudad de Panamá, Panama, Panama; 5 Department of Public and Ecosystem Health, Cornell University, Ithaca, NY, United States; 6 Dirección del Laboratorio Nacional de Salud, Ministerio de Salud Pública y Asistencia Social, Ciudad de Guatemala, Villa Nueva, Guatemala; 7 General Coordination of Public Health Laboratories, Health and Environmental Surveillance Secretariat, Ministry of Health of Brazil, Brasilia, Brazil; 8 Centre for Epidemic Response Innovation (CERI), School for Data Science and Computational Thinking, Stellenbosch University, Stellenbosch, South Africa; 9 Collaboratory for Emerging Infectious Diseases and Response, Tufts University, Boston, MA, United States; 10 Division of Geographic Medicine and Infectious Diseases, Tufts Medical Center, Boston, MA, United States; 11 Center for Global Health and Tropical Medicine, Instituto de Higiene e Medicina Tropical, Universidade Nova de Lisboa, Lisbon, Portugal; 12 Blood Center of Ribeirão Preto, São Paulo, Brazil; 13 Medical School of Ribeirão Preto, São Paulo, Brazil; 14 Universidade Federal de Mato Grosso do Sul, Campo Grande, Mato Grosso do Sul, Brazil; 15 Panamerican Health Organization, World Health Organization, Brasilia, Brazil; 16 Central Public Health Laboratory, Mato Grosso State Health Department, Campo Grande, Brazil; 17 Panamerican Health Organization, World Health Organization, Guatemala, Guatemala; 18 Nicaragua Ministry of Health, Managua, Nicaragua; 19 Labor - Health Supply, São Paulo, Brazil; 20 Institute of Microbiology and Immunology, University of Belgrade Faculty of Medicine, Belgrade, Serbia; 21 Department of Environmental and Occupational Health Sciences, University of Washington, Seattle, WA, United States; 22 Department of Global Health, University of Washington, Seattle, WA, United States; 23 Department of Family Medicine, University of Washington, Seattle, WA, United States; 24 Department of Medicine, University of Washington, Seattle, WA, United States; 25 Division of Allergy and Infectious Disease, University of Washington, Seattle, WA, United States; 26 Center for One Health Research, University of Washington, Seattle, WA, United States

**Keywords:** COVID-19, genomic epidemiology, pandemic preparedness, transdisciplinary training, systems thinking, capacity building

## Abstract

During the COVID-19 pandemic, the emergence and detection of SARS-CoV-2 variants of concern highlighted the pivotal role of genomic epidemiology in public health decision and policy making. The pandemic also revealed a need for genomic epidemiologists to effectively engage with a broader audience, including policymakers and the public. To address this need, we introduced a new transdisciplinary training workshop, “From Trees to Public Health Policy,” at the Virus Evolution and Molecular Epidemiology (VEME) 2022 workshop in Panama City, designed to foster an enhanced understanding of the current challenges translating the results of genomic epidemiology into public health decision-making. Transdisciplinarity provides a collaborative problem-solving approach that integrates multiple disciplines and stakeholders to address complex and difficult to define problems, often referred to as “wicked problems.” We argue that training in transdisciplinary approaches within genomic epidemiology will more effectively prevent, prepare for, and mitigate future pandemic risks. Here, we introduce this new module and methodology, along with detailed output, reflections on its implementation and outcomes, as well as areas for improvement for future iterations of the workshop. Workshop participants (*n* = 19) were selected across multiple levels of the genomic surveillance-to-policy continuum and split into two groups to engage in a four-day transdisciplinary learning process using the Designing Feasible Futures Framework (DF3). Through iterative exercises, participants mapped the complexity of the genomic surveillance for public health system, identifying leverage points for intervention, multi-sectoral stakeholders involved, and exploring futures scenarios following the proposed interventions. The two working groups developed complementary approaches: one prioritizing data infrastructure in low-resource settings, and the other emphasizing community trust and engagement. Evaluation of the workshop included pre- and post-workshop questionnaires, group self-evaluations, and confidential feedback. Group evaluations revealed varying levels of happiness and frustration throughout the iterative activities, and pre-post assessments showed statistically significant improvements in participants’ self-reported confidence in understanding wicked problems, systems thinking, and transdisciplinary collaboration. The proportion of participants endorsing shared decision-making across scientists, policymakers, and other stakeholders increased from 50 to 82%. Overall, this pilot workshop showed the feasibility and value of transdisciplinary training for practitioners of genomic epidemiology.

## 1 Introduction

Genomic epidemiology of infectious disease has experienced a large shift in prominence and importance over the last couple of years, fueled by the COVID-19 pandemic and the rapid expansion of capacity for pathogen genomic sequencing and analysis. At its core, the field focuses on using viral or bacterial genome sequences to characterize an underlying pathogen population, tracing its evolution and transmission dynamics (1–3). While new sequencing technologies have reduced costs over the past two decades, making genomic epidemiological studies more accessible (4), the development of better yet more complex methods has resulted in the requirement of even more specialized expertise in bioinformatics, evolutionary biology, and statistical phylogenetics (5). The scale of currently available data is unprecedented. As of September 2025, over 17 million SARS-CoV-2 sequences have been generated and shared by more than 190 countries around the world (6). This has allowed for tracking the evolution of SARS-CoV-2 across the full spectrum of scales ranging from within-host (7) and transmission within a single hospital (8), to the municipal (9), national (10), and cross-national scales (11). The most visible application of this capacity has been monitoring, identifying and characterizing emerging SARS-CoV-2 variants of concern or interest (VOC/I) (12). Such variants refer viral strains that contain a constellation of mutations that result in the virus being more transmissible, virulent, or immune-evasive, which may affect rates of community transmission or mortality (13). To date, a total of 15 VOCs and VOIs have been classified by WHO, each with a corresponding Greek letter name (14). Monitoring variants for public health decision-making thrust the once obscure field of genomic epidemiology into prominence as stakeholders sought answers about the virus and its emerging mutations.

The increased visibility made it clear that partnerships between academics, public health practitioners and the broader policy sector were required to effectively track the pandemic and inform intervention strategies. However, the diversity in expertise and mental models across sectors challenges effective collaboration. Policymakers and public health officials often lack the expertise in evolutionary virology and phylogenetics (often found in academic departments) required to interpret genomic analyses. Conversely, evolutionary virologists and molecular epidemiologists may not fully understand the societal and economic concerns driving policy decisions. Moreover, these groups can operate under differing and sometimes opposing incentives, as crisis conditions demand timely decisions based on potentially suboptimal data. This complex interplay of expertise, conflicting incentives, urgency, and societal impacts positions pandemic preparedness and response as a ‘wicked problem’, a problem that is inherently resistant to a clear definition and agreed solutions (15). Transdisciplinarity, the highest degree of collaboration between academic and non-academic disciplines and knowledge systems (16), emerges as a promising approach to tackle such challenges, as it requires common goal setting across all stakeholders involved. However, given the predominance of discipline-centric approaches, the implementation of transdisciplinary approaches requires dedicated training and educational initiatives to shift the existing paradigms toward a more integrative problem-solving approach.

For nearly three decades, the international Bioinformatics Workshop on Virus Evolution and Molecular Epidemiology (VEME) has trained scientists worldwide in genomic epidemiology (^1^^,^^2^), building a global network equipped for outbreak response. Here, we describe the introduction of a new transdisciplinary training module at the VEME workshop, designed specifically to identify and bridge gaps between scientists and policymakers in the context of genomic epidemiology. The first instance of this new module, called “From Trees to Public Health Policy,” took place in August 2022 in Panama City at the workshop’s 26th edition.

## 2 Pedagogical framework

The new transdisciplinary learning module at VEME is structured based on the Designing Feasible Futures Framework (DF3) developed by the KU Leuven’s Institute for the Future (17). This iterative framework consists of four modules that aim to provide a systematic approach to facilitate transdisciplinary processes: (a) problem framing, (b) complexity analysis, (c) multi-level stakeholder involvement, and (d) designing feasible futures (Supplementary Data Sheet 1; Figure 4). More specifically, each of the modules aims to (a) leverage the diversity in the viewpoints to establish a common goal and understanding, (b) deepen the team’s understanding by contextualizing the problem in a systems setting, (c) identify a broad set of stakeholders and actors relevant to the problem, and (d) co-create a complexity informed vision of the future mapping desired targets and actions. Sequential progression through these modules generates three knowledge types: *systems*, *target*, and *transformational* knowledge (18). Systems knowledge refers to understanding the complexity of the problem at hand. In the context of genomic epidemiology, this means deeply analyzing the interconnected factors that influence disease spread, surveillance and policy decisions. Target knowledge focuses on defining desired future states and core values that guide decision making. Examples in the pandemic context include minimizing disease transmission, ensuring equitable resource distribution, or developing more robust surveillance systems. Transformational knowledge identifies steps bridging present and future states, requiring actor identification, challenge assessment, and strategic planning. For a more comprehensive overview of the framework, we refer readers to Supplementary Data Sheet 1.

## 3 Learning environment

The DF3 was fundamentally designed to be an iterative transdisciplinary methodology, allowing for multiple refinement cycles. However, due to practical time constraints, the VEME workshop aimed to introduce participants to a single, comprehensive iteration of the framework over the course of 4 days. The workshops consisted of both lectures by experts and practical sessions. Each morning began with theoretical and topical lectures on transdisciplinarity and genomic surveillance, delivered by domain experts. These lectures preceded the facilitator-led practical sessions, which ensured a shared baseline understanding from which new collaborative knowledge could be built upon^3^. An overview of the pedagogical objectives and methodologies used in each day can be seen in Table 1.

**Table 1:** Overview of the ‘From trees to Public Health Policy’ workshops’ framework, methodology and pedagogical goals.

Module	Day 1: Framing	Day 2: Complexity	Day 3: Actors	Day 4: Futures	Every day: Teambuilding
Methodology	Joint Theory of Change	Systems mapping (causal loop diagram) and leverage point analysis	Mapping levels of engagement of stakeholders	Futures Wheel based on leverage point analysis and proposed action	Convergent-divergent thinking facilitating co-creation
Tool	WHY? Motivation HOW? Process WHAT? Outcomes	PESTEL+H analysis (Political, Economic, Social, Technological, Environmental, Legal and Health factors), followed by group systems mapping exercised based on these factors	Actor constellation exercise	Define first, second and third order consequences of the action	Safe-space, brainstorming, Active listening, facilitators ask reflective questions rather than correcting answers or directing the workshop
Purpose	Subsystem boundaries and vision alignment	Systems knowledge and leverage points	Actors to engage in the action	Scenarios and associated interventions	Trust to co-create

Furthermore, participants were presented with a challenge document prior to the start of the workshop to prime the transdisciplinary process. This document introduced a case study focusing on the discovery of the Omicron variant and the resulting travel bans implemented in response to its first detection in South Africa (Supplementary Data Sheet 2). Additionally, all participants provided verbal consent for documenting and potentially publishing their workshop experiences, as detailed in this paper.

Participants were divided into two subgroups of approximately 10 people, each composed to maximize the diversity of mental models. They were intentionally selected to maximize diversity in both technical expertise and professional experience, ensuring that a broad range of perspectives relevant to genomic epidemiology and public health decision-making were represented. As part of the workshop assessment, participants were asked to indicate their professional background(s). In total, 11 reported a background in public health or medicine, 7 in genetics or bioinformatics, 3 were policy makers, and one reported microbiology while another reported infectious disease modeling. To guarantee inclusivity and facilitate full participation, a Spanish to English translator was provided for one group, which included most of the participants from Latin America (Group ES). The other group conducted sessions entirely in English, comprising participants from diverse global locations (Group EN). Each team was supported by two coaches experienced in the DF3 methodology, with one also having subject matter expertise. Facilitators shared responsibilities for process guidance, team cohesion, timekeeping, and ensuring the generation of outputs from each exercise. Team building was prioritized throughout the entirety of the workshop.

### 3.1 Day 1

Day one of the workshop focused on building team unity and engaging in joint problem framing (19, 20). The initial icebreaker had participants introduce themselves by drawing country landmarks or foods on post-its placed on a world map. This was done to foster connection between the participants and highlight the diversity of backgrounds present. Once the groups joined their coaches, a *safe space* exercise was done to ensure a workshop where participants could speak freely and engage in open, inclusive, and respectful dialog. The group collaboratively defined the principles of their safe space, documenting these guidelines on a shared flipchart that was signed by all participants. Potential issues that might arise during the training regarding consent, confidentiality and COVID-safety were also discussed, with the signed document serving as a daily reminder of the group’s commitment to a productive and respectful transdisciplinary process.

To *frame* the public health challenge, participants engaged with the Theory of Change (ToC) methodology (21), a framework for understanding and proposing systemic change. ToC serves as both an analytical process and a living document that explores how change happens in a complex system that interacts with diverse stakeholders (21, 22). Prior to the start of the workshop, each attendee was asked to draft an individual ToC, addressing four reflective questions relating to the challenge document: (a) *why* is change needed, (b) *how* does change happen and *who* do we need for this change to happen, (c) *what steps* do we need to take, and (d) *what outcome* do we expect. The goal of this individual exercise was to facilitate personal reflection and allow the participants to form and express their opinions without potential group influence. Subsequently, participants within each group formulated a joint ToC by identifying the common themes of their individual reflections. This collaborative process served two main functions: aligning diverse visions and establishing a shared focal point that defined the boundaries of the system they would focus on for the remainder of the workshop.

### 3.2 Day 2

Building upon the problem framing, the teams collaboratively developed a systems map of the problem using causal loop diagrams (CLDs) to analyze the *complexity* of their system in question (23). CLDs are built linking factors that represent variables in the system capable of increasing or decreasing, with connections depicting causal relationships with positive and negative interactions. This methodology allowed participants to transport their challenge from the conceptual realm to a tangible system, allowing for a more nuanced understanding of interactions and moving beyond linear problem-solving.

The selection of factors was done using the PESTEL+H framework (encompassing Political, Economic, Social, Technological, Environmental, Legal and Health dimensions) to promote holistic and multidisciplinary perspectives (Supplementary Data Sheet 3; Supplementary Figure S1–3). Our framework differs slightly from the PESTEL framework used traditionally in organizational analysis (24) in that a dedicated category of Health was added to better fit the context of the workshop. The team collectively selected the most important factors and placed them on a map along with their positive and negative interactions to create a visual representation of the complexity of the system. By identifying driving factors and reinforcing feedback loops, participants explored potential future scenarios and leverage points that could be used to design potential interventions.

### 3.3 Day 3

Building on the identification of potential leverage points and a desired intervention, a stakeholder constellation exercise (25) was conducted to identify the actors necessary for designing an intervention at a leverage point. The teams chose one potential leverage point to investigate. The exercise began with two guiding questions: “Who can influence or has influenced this potential leverage point?” and “Who is or was involved in this potential leverage point?”

Next, actors were arranged in concentric circles around the central inquiry, reflecting their level of influence and importance (Supplementary Figure S2). Key stakeholders placed closer to the center signified greater relevance and impact on the leverage point. Using the quintuple helix of innovation framework (26), which ensures representation from academia, industry, government, civil society, and the environment/natural ecosystems, each participant appointed relevant stakeholders around the central inquiry based on their legitimacy, power, and urgency. Following this individual contribution, feedback was provided, facilitating a group discussion, and moving the position of each actor across different levels until consensus was reached. The different levels of actors in the graph roughly represent the different levels of engagement in a multi-level stakeholder engagement approach (27). The resulting map illustrated a hierarchy of various stakeholders across diverse fields and provided an understanding of their interrelations and collective impact within the system.

### 3.4 Day 4

On the final day of the training, participants engaged in a Futures Wheel exercise (28), building on the joint Theory of Change (ToC) and the leverage points identified in previous sessions. This exercise aimed to explore the potential consequences of actions related to their selected leverage point.

Participants began by identifying direct (positive or negative) consequences associated with the chosen leverage point. Subsequently, they analyzed secondary consequences, followed by third-order effects, creating a multi-layered wheel that visually represented these outcomes (Supplementary Figure S3). By iterating through these levels of consequence, participants gained insights into the cascading effects that actions can have within the system. Such insights can aid in the development of future scenarios, encouraging critical thinking about long-term implications of their interventions.

### 3.5 Daily reporting and evaluation

Daily group reporting and self-evaluation exercises were implemented to encourage team reflection and inter-team learning. At the end of each day, teams reflected on the workshop content and their emotional experiences, preparing a report for the other team. Content reporting was flexible and left to each team’s discretion, while team dynamics were depicted by a happiness-frustration curve plotted against a timeline. Teams selected events for commentary on the X-axis (using the workshop schedule as a guide) and determined their own Y-axis scales. From the second morning onward and prior to lectures and practical sessions, teams presented oral reports summarizing the previous day’s output and reflections. Two rotating participants per team presented: one on outputs, the other on happiness and frustration.

Initially, an independent evaluator was assigned to observe the process; however, due to the workshop’s interactive nature, this evaluator ultimately became a participant in one of the groups. Individual evaluations consisted of a pre-test and post-test questionnaire, in which participants answered five questions assessing their confidence and familiarity with the contents of the workshop. Responses were recorded on a 10-point Likert scale ranging from strongly disagree to strongly agree. Additionally, participants were invited to express their views on who should primarily influence policy decisions regarding emerging pathogens by positioning their preferences within a simplex plot of politicians, scientists, and stakeholders. Participants were also given the opportunity to provide confidential written feedback at the end of the workshop.

### 3.6 Data collection

Data for this article was collected through focus group discussions. The study received ethical approval, and all participants gave written informed consent. Personal data were processed in accordance with the General Data Protection Regulation (GDPR) standards to ensure data protection.

## 4 Results

The iterative nature of transdisciplinary research under the DF3 means that a 4-day workshop is not sufficient to produce comprehensive outcomes. Rather, the primary purpose of the workshop was to introduce participants to the fundamental concepts, theories, and methodologies involved in transdisciplinary research. Additionally, it provided a platform for participants to engage with the collaborative processes required when working with a diverse team of scientists and policymakers. Given the workshop’s limited duration and introductory focus, this results section outlines the output and experiences at each stage of the DF3 as presented by the teams, without extensive fact-checking or interpretation of the data presented.

### 4.1 Day 1

As previously explained, the first day was centered around each group drafting a joint ToC on the challenge they aimed to tackle throughout the workshop. Participants used the challenge document as a guide but were also given the freedom to shift their focus to a different problem within the system of genomic surveillance and policy decision-making. Due to the diverse backgrounds present in each team, the resulting joint ToCs varied significantly.

In response to the question ‘why is change needed’, Group EN linked the lack of pandemic preparedness with the wickedness of the problem, and advocated for a broader, more holistic approach since policy decisions based on the molecular epidemiology of SARS-CoV-2 are a *“public problem, not just a public health problem.”*

Group ES highlighted the importance of bridging gaps among scientists, policymakers, and the public to foster global solidarity. The group identified trust as one of the key elements: “*Underlying this is trust, and we spoke a lot about trust. Trust in government, trust in scientists.*” The group also highlighted the role of political leadership in recognizing the community and being honest with a global vision.

Both groups agreed on the axes of change, identifying infrastructure, human capacity, and global partnerships as key dimensions. Group EN proposed a three-pronged approach focusing on infrastructure for proactive responses, training, and international cooperation, while Group ES stressed the importance of education and the empathetic communication of public health messages. As one member noted, “*Empathy is critical, reflected in culturally sensitive messaging*.”

In their “what actions” section, both groups emphasized fostering inclusive dialog and building personal relationships. Group EN suggested workshops and simulations with stakeholders, while Group ES explicitly mentioned inclusive dialog as foundational. Both groups also recognized the need for resources toward training and technology to enhance pandemic response capabilities. Additionally, both groups stressed the importance of engaging with communities and listening to them in an empathetic and culturally sensitive manner. Context specific communication is key, as noted by a member of Group EN “*Communication needs to happen differently when there is an emergency versus a normal situation*.”

Distinct outcomes emerged in the “what outcomes” section. Group EN struggled to articulate a consensus statement on expected outcomes and presented individual goals instead. Conversely, Group ES clearly identified aspirations for personal growth and a transformative understanding of transdisciplinary approaches, highlighting their commitment to continued collaboration through “*a bond between team members,*” and the opportunity to “*acquire knowledge from other individuals’ life experiences and perspectives.*”

**Figure 1:**
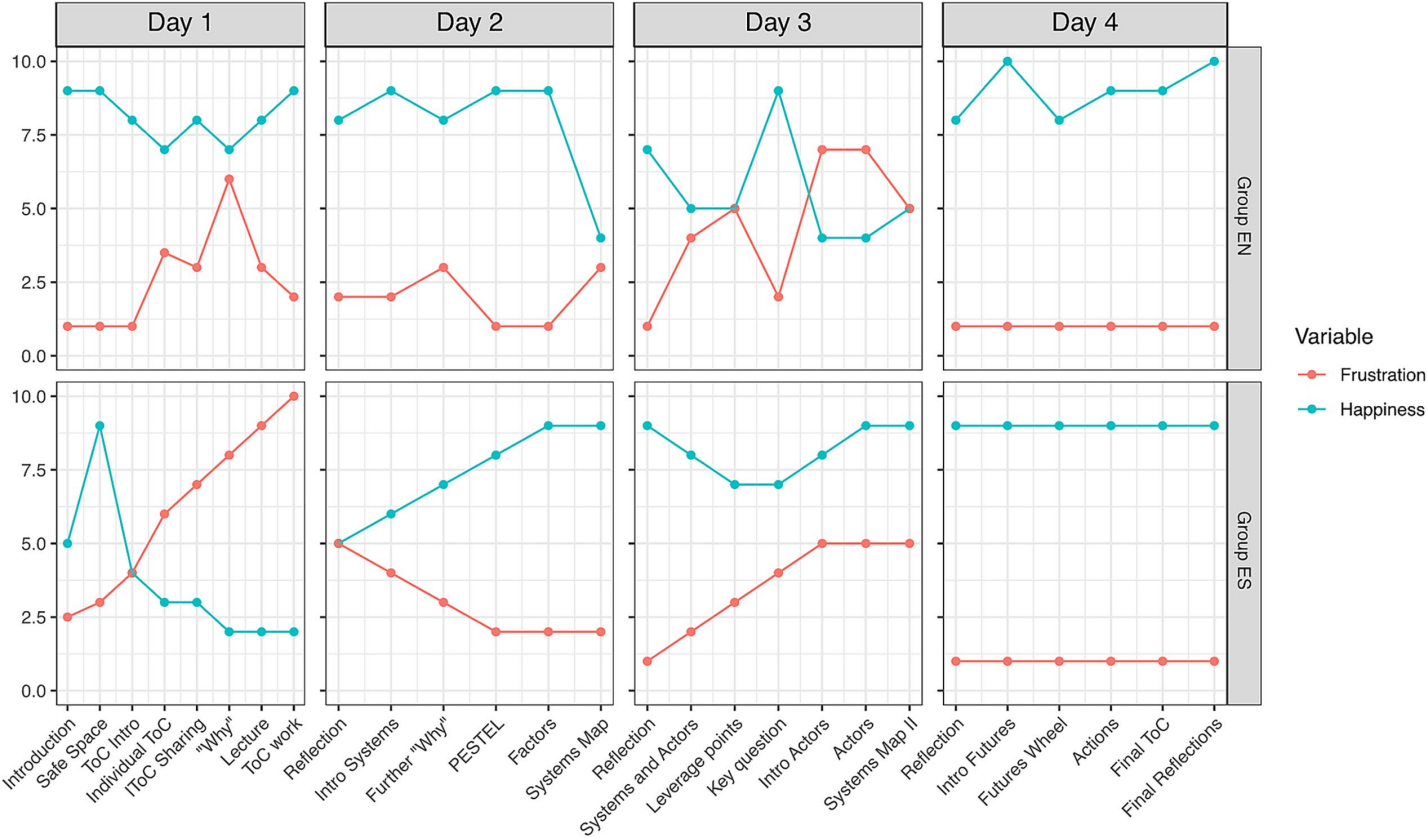
Happiness and frustration curves for Group EN and Group ES over the 4-day workshop. The horizontal axis displays the sequence of activities and events chronologically throughout the workshop. The vertical axis represents the team’s collectively agreed upon levels of happiness (blue curve) and frustration (red curve) experienced during each activity. This visualization captures the group’s emotional journey and engagement fluctuations, providing insight into the collective experience at different stages of the workshop process.

Despite the thematic commonalities, the group evaluations revealed some differences in the happiness and frustration experienced by each team. Group EN reported surprise and illumination during discussions, contributing to increased happiness, although some frustrations emerged due to team dynamics (Figure 1). Group ES experienced a consistent increase in frustration throughout the day (Figure 1), which they attributed to language barriers and an unclear schedule.

### 4.2 Day 2

**Figure 2:**
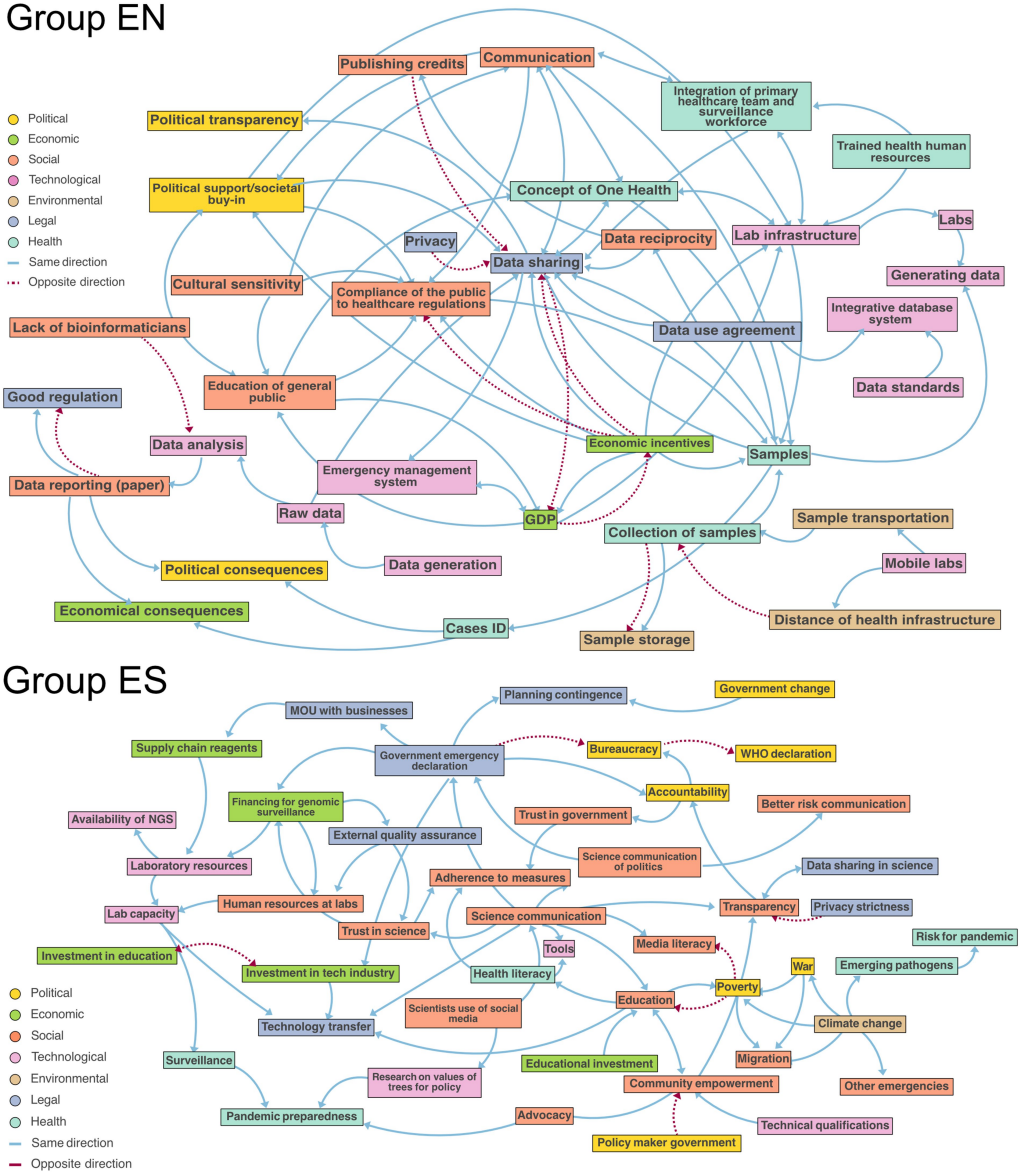
Causal loop diagram mapping Group EN and Group ES’s subsystem of the wicked problem. Each factor has been colored according to its PESTEL+H classification, with blue arrows denoting same direction causality (i.e., an increase/decrease in the value of a factor results in a change in the same direction) and red dashed arrows opposite causality (an increase/decrease in the value of a factor results in change in the opposite direction).

On the second day, participants built upon their Theory of Change by creating causal loop diagrams (CLDs) to analyze subsystems related to their chosen wicked problem. Group EN focused in low- and middle-income countries, which they identified as a priority from their ToC. They identified key factors such as data sharing, data reciprocity, communication, economic incentives, One Health, and public education as crucial to their system (Figure 2). Although the group primarily focused on data-related factors, they acknowledged the need for a multifaceted approach, highlighting the importance of collaboration, public awareness, and technological integration. While they easily identified positive correlations, such as the relationship between laboratory infrastructure and data sharing, they struggled to pinpoint negative correlations. Additionally, they noted that the influence of factors could change over time, adding complexity to their analysis. As the day progressed, fatigue set in, leading to increasing challenges and frustration in assembling their systems map (Figure 1).

In contrast, Group ES adopted a different strategy, prioritizing the social dimensions of pandemic response (Figure 2). One participant remarked, “*We chose as an outcome to work on reaching better harmony between scientists, decision makers and the public*.” They initially addressed a broad topic but narrowed their focus to “understanding the values of trees for decision-making” specific to their field. Their systems map included laboratory components, like resources and science communication, alongside governmental factors such as trust and transparency. Their CLD showed strong alignment with their initial “why” statement, which aimed to “improve comprehensive pandemic response through better integration of laboratory diagnostics and epidemiological surveillance.” This connection between their goals and the resulting map pleasantly surprised the group, leading to positive experiences and an overall increase in collective happiness throughout the day (Figure 1).

### 4.3 Day 3

Participants began Day 3 by identifying a leverage point within their system, which would serve as a focal point for subsequent activities. After determining the leverage point, teams moved on to an actor constellation mapping exercise, which aimed to identify stakeholders and societal actors who could influence or be affected by actions taken at this leverage point.

In the case of Group EN, finding a leverage point to pose the central question was a major point of frustration, yet the outcome was ultimately rewarding. One of the participants remarked, *“Zooming in the systems map [and] identifying our leverage point, poses substantial frustration to the group. But then, the moment when the question was finally defined, was both the moment of surprise and the moment of hope.”* This transition is reflected in their happiness-frustration curve, showing a marked decrease in frustration and an increase in happiness after agreeing on the leverage point (Figure 1). Group ES also encountered challenges as they transitioned from the systems map, with frustration increasing throughout the day (Figure 1).

**Figure 3:**
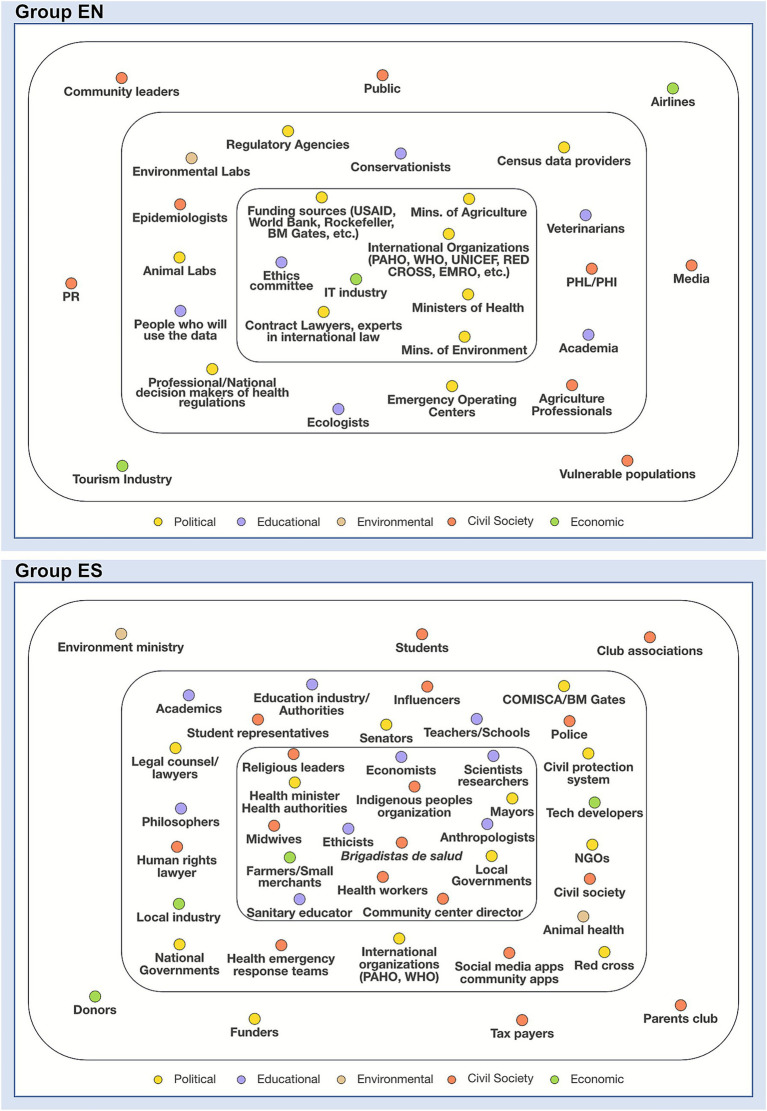
Actor Constellation for groups ES and EN. This diagram presents the results of each group’s actor constellation exercise, a visual mapping of stakeholders relevant to their respective problem. Each colored dot represents a specific actor or stakeholder group, with their position in the diagram reflecting their level of proximity and influence on the central issue. The color of each actor corresponds to a domain within the quintuple helix model of innovation: Political (yellow), Educational (purple), Environmental (brown), Civil Society (orange), and Economic (green).

The focus of each group diverged significantly. Group EN aimed to identify actors for establishing an international technical and legal framework to enhance data sharing in low- and middle-income countries within a One Health context. Conversely, Group ES sought to identify actors for co-designing strategies to enhance community commitment to public health responses (Figure 3), referring to these as a “compass for community engagement in pandemic response.”

The differences in focus were also reflected in the actors identified. Group EN included fewer stakeholders, such as government ministers, international funding organizations, and industry representatives, particularly emphasizing the technology sector (Figure 3). Meanwhile, Group ES’s actor constellation included a broader range of participants, from government authorities to scientists and community members (Figure 3). Inclusion criteria for Group ES centered on “*people that work and live directly with communities*,” with particular emphasis on the essential role of midwives. One member noted, “*In Latin America, many births happen in communities rather than hospitals. Midwives are trusted figures, lending legitimacy to public health initiatives*.”

Overall, both teams reported challenges with this exercise, leading to increased frustration throughout the day (Figure 1). Group EN struggled with the complexity of considering multiple actor categories, stating, “*we agreed that this was a set point of frustration… We got to the point where we had to make a break before proceeding.*” Similarly, Group ES found it difficult to visualize and decide on inclusions in their mapping: “*It is really difficult to visualize with a graphic like that, who one has to include and leave out.*”

### 4.4 Day 4

The final day of the workshop consisted of the Futures stage of the DF3. Here, the goal was for each team to integrate the insights gained from the previous days to construct a vision for the future, along with plausible scenarios. Building on the leverage points identified the day before, teams decided on a potential intervention at this leverage point, and, through a Futures Wheel exercise, envisioned both positive and negative first, second and third-order consequences of implementing this intervention, promoting a deeper understanding of potential implications.

**Figure 4:**
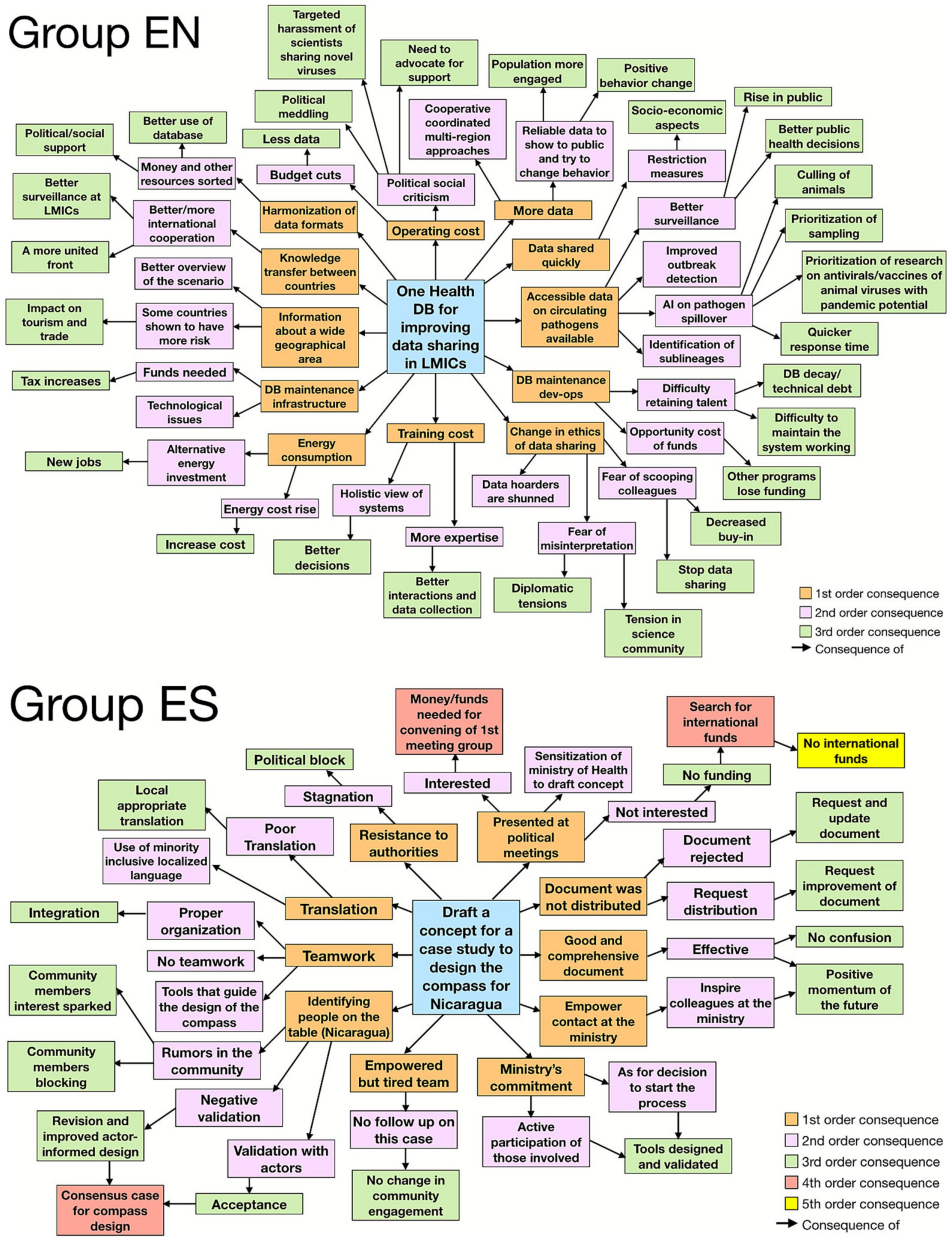
Futures Wheel exercise from groups ES and EN. On each wheel, the central node represents the proposed intervention. Each concentric ring of colored nodes corresponds to increasing orders of consequences: orange nodes indicate first-order consequences, blue pink represent second-order consequences, green nodes show third-order consequences, red nodes indicate fourth-order consequences and yellow nodes fifth-order consequences. This visual mapping illustrates the potential cascading effects of the proposed intervention.

Due to time constraints, the teams were unable to formally present their results. Therefore, this section primarily highlights their experiences during the exercise. The Futures Wheel outputs for Groups EN and ES can be seen in Figure 4. Overall, both teams reported positive feelings, reflected in consistently low levels of frustration throughout the day (Figure 1). A participant from Group EN observed: “*things were really starting to come together*.” Similarly, Group ES members expressed optimism: “*We really had very low frustration throughout the day. I’ll add that we are really very hopeful for the future because we really are taking away the change*.”

The workshop concluded with participants sharing their overall experiences from the 4 days. Feedback was overwhelmingly positive, with many finding the workshop methodology innovative and enriching. One participant commented the workshop was “*far surpassing my expectations because, I think not only did we come away with a new way of approaching problems, we came away with a new way of understanding one another*.” The diversity of participants and ideas was particularly valued: “*someone would put something on the* Table 1 *would never have thought about and that was so enriching, and I really liked it.”* Participants also appreciated how the workshop made them feel empowered: “*it’s the participant who brings the rule […] I go back with a new way of thinking and a new way of working for the future.”*

### 4.5 Evaluation and feedback

We refer the readers to the evaluation report for a more detailed account of the survey results (Supplementary Data Sheet 4). Briefly, the results can be summarized as follows. Overall, the group evaluations showed qualitatively different experiences in happiness and frustration between the two groups. The groups differ in terms of which stages they found more frustrating and not, with Day 1 and Day 3 presenting the most challenges. By Day 4 however, the levels of frustration were low and happiness high for both groups.

The individual evaluations showed that participants were initially somewhat familiar with the concepts presented in the workshop but reported a statistically significant increase in confidence in their understanding of wicked problems, systems thinking and transdisciplinarity. Furthermore, the proportion of participants that thought that decision making should be shared across politicians, scientists, and stakeholders increased from 50 to 82%. However, this trend was not statistically significant.

Results from the anonymous feedback indicated that participants found value in the overall course structure, innovative methodology, instructor knowledge, and lecture content. Participants specifically noted the well-defined milestones, the effective blend of theory and practice, and the valuable transdisciplinary experience gained from interacting with a diverse group of individuals. However, some participants initially found the methods confusing and the workshop pace too intense. Time constraints were a recurrent theme, with multiple participants indicating that there was insufficient time allocated for activities, affecting their ability to thoroughly engage with the content and complete tasks effectively. Suggestions for improvement included providing pre-workshop materials for self-study, adjusting the course length and time allocated for individual exercises, incorporating exercises focused on group dynamics, organizing group social activities, and establishing a stronger connection to bioinformatics.

## 5 Discussion

The creation of the VEME From Trees to Public Health Policy workshop represents a critical response to the complex challenges posed by our current era of accelerated environmental and social change. In this context, traditional approaches to pandemic preparedness have become increasingly inadequate (29). Transdisciplinary approaches have emerged in response as a new paradigm for addressing such complex global health challenges, offering a promising alternative to siloed research and strategies, particularly in the context of climate change and infectious diseases (30).

While transdisciplinary initiatives are still nascent in the realm of pandemic preparedness, they have shown significant potential (31). For instance, unique applications have been demonstrated in contexts like Alaska, where in 2023 circumpolar researchers and indigenous partners collaborated to identify pandemic research priorities, integrating indigenous knowledge and holistic understanding of regional contexts (32). Similarly, researchers have explored transdisciplinary frameworks for designing innovative pandemic preparedness tools, such as “serious game” exercises (33).

The complexities of the COVID-19 pandemic highlighted significant gaps in the translation of scientific expertise into policy (34). Our experiences during the pandemic have shown that our current genomic surveillance approach requires a fundamental reimagining to achieve meaningful societal impact. As such, the VEME From Trees to Public Health Policy workshop module was organized to introduce transdisciplinary thinking to scientists, public health practitioners, and policymakers working in the field. We believe that reimagining genomic epidemiology and surveillance requires not just new methodologies, but a fundamental transformation in our thinking paradigms, and that this workshop represents a significant step toward bridging these gaps.

The value of conducting a workshop with such a diverse team in this field cannot be overstated. By bringing together participants from diverse backgrounds, the workshop facilitated the exchange of knowledge, perspectives, and experiences, fostering a deeper understanding of the complex nature of emerging infectious diseases. This diversity allowed participants to gain insight into the challenges faced by different sectors and to appreciate the importance of a holistic approach. In addition, the developed DF3 framework offers a methodological blueprint for future transdisciplinary research. Our aim is to foster the creation of spaces for genuine dialog and knowledge co-creation, where we can develop more nuanced, effective strategies for preparing against emerging health challenges.

As organizers, we have also learned valuable lessons from conducting this workshop, which was the first of its kind in the field. We recognize the need to better define the challenge document, finding that narrowing the scope would be more effective than a broad approach. Additionally, we observed that the transdisciplinary process ideally requires a team that has gathered by mutual choice, a limitation in our current format, as participants were distributed by background and expertise without prior connection. The overrepresentation of participants from science and academia (the historical audience for the VEME workshop), is another weakness. An unexpected challenge was the language barrier between participants, highlighting the importance of linguistic accessibility in fostering effective transdisciplinary research. These factors made it difficult for the teams to continue their work after the workshop ended. Thus, the format and impact of the workshop, as well as the actionability of the outcomes, remain as areas for improvement. To address these issues, we aim to refine the workshop in future editions, for it to lead to a meaningful and lasting impact that can result in policy implementations. It is our hope that the long-term potential impact of this workshop extends far beyond its immediate outcomes. By introducing transdisciplinary approaches to genomic surveillance and public health policy, we aim to lay the groundwork for a paradigm shift in how we approach complex global health challenges within the field of genomic epidemiology.

Despite these challenges, participants expressed strong excitement and willingness to continue with the work initiated at VEME. The workshop’s inspirational value was clear in the immediate actions of participants: one professor redesigned a One Health class using this framework, another planned to introduce similar coursework, and a public health researcher applied for transdisciplinary research grants. We view this workshop as a fertile ground for creating change—a first step toward changing how we approach pandemic preparedness. As more professionals become versed in transdisciplinary thinking, we anticipate more comprehensive policy decisions that balance scientific evidence with societal needs and constraints. Furthermore, the workshop’s emphasis on collaboration may catalyze new innovations, potentially resulting in the development of novel surveillance and intervention strategies.

Ultimately, we believe this workshop represents a crucial first step toward transdisciplinary collaboration in the field of emerging infectious diseases and genomic surveillance. While the workshop is not yet fully refined for this purpose, the enthusiasm and positive outcomes generated by participants indicate its significant potential. As participants and organizers, we remain committed to refining our approach, bridging disciplinary divides, and developing more holistic strategies for genomic surveillance that genuinely serve societal needs.

## Data Availability

The raw data supporting the conclusions of this article will be made available by the authors, without undue reservation.

## Footnotes

1
https://rega.kuleuven.be/cev/veme-workshop/2022

2
https://veme.climade.health

3
https://veme.climade.health/veme2022/
