# Supplementary material for: Transdisciplinary training to address challenges in genomic epidemiology of infectious diseases
Source: Front Public Health. 2025 Dec 17;13:1713182. doi: 10.3389/fpubh.2025.1713182 (PMC12753939; doi:10.3389/fpubh.2025.1713182)
Supplement: Supplementary file 1 [file Data_Sheet_1.pdf]

A transdisciplinary framework by the Leuven Institute for the Future: The Designing Feasible Futures Framework (DF3)

Anne-Mieke Vandamme[1,2,3] & Jorge Nova[1,2]

1. KU Leuven, Institute for the Future, Leuven, Belgium
2. KU Leuven, Department of Microbiology, Immunology and Transplantation, Rega Institute for Medical Research, Clinical and Epidemiological Virology, Leuven, Belgium
3. Center for Global Health and Tropical Medicine, Unidade de Microbiologia, Instituto de Higiene e Medicina Tropical, Universidade Nova de Lisboa, Lisbon, Portugal

Corresponding author: Anne-Mieke Vandamme (annemie.vandamme@kuleuven.be)

Table of Contents

Background on the DF3 framework *1*

The need for transdisciplinary frameworks *4*

The need for the DF3 framework 5

The DF3 framework outline 5

Essential concepts that need to be understood and addressed *7*

Wicked problems 8

Transdisciplinarity 11

Complexity 11

Three types of knowledge 11

Background on the DF3 framework

The Leuven Institute for the Future[1] was launched in 2016 with the effort of three professors from different groups of sciences (Humanities and Social sciences, Science, Engineering and Technology, and Biomedical Sciences) and a business manager with the aim to foster Transdisciplinary research at KU Leuven.

1 https://rega.kuleuven.be/if

The inspiration for the methodology used at the Institute for the future was the quintuple helix of innovation[2,3], whereby the socio-ecological environment and the concerns around reaching the 17 SDGs are a driving force of innovation, in addition to the already known 4 actors from the quadruple helix of innovation: academia, industry, civil society, government. Figure 1 shows the outline of the quintuple helix model of innovation the way it is applied at the Institute for the Future. Challenges are collected from 4 types of actors inspired by improving the socio-ecological environment as outlined by the 17 SDG's, which is considered the 5th "actor". The challenges are incubated by small teams (5 to 20 individuals), paying attention to the diversity of the team that is co-creating knowledge within the ethical framework of the SDGs. The efforts are geared towards solution-oriented thinking, responsible research and innovation, and delivering insights and foresights. The results can be inspiration for new research, businesses, policies, improvements towards the SDGs, or can be of immediate value by transforming the world view of the team or by the production of output such as movies, publications.

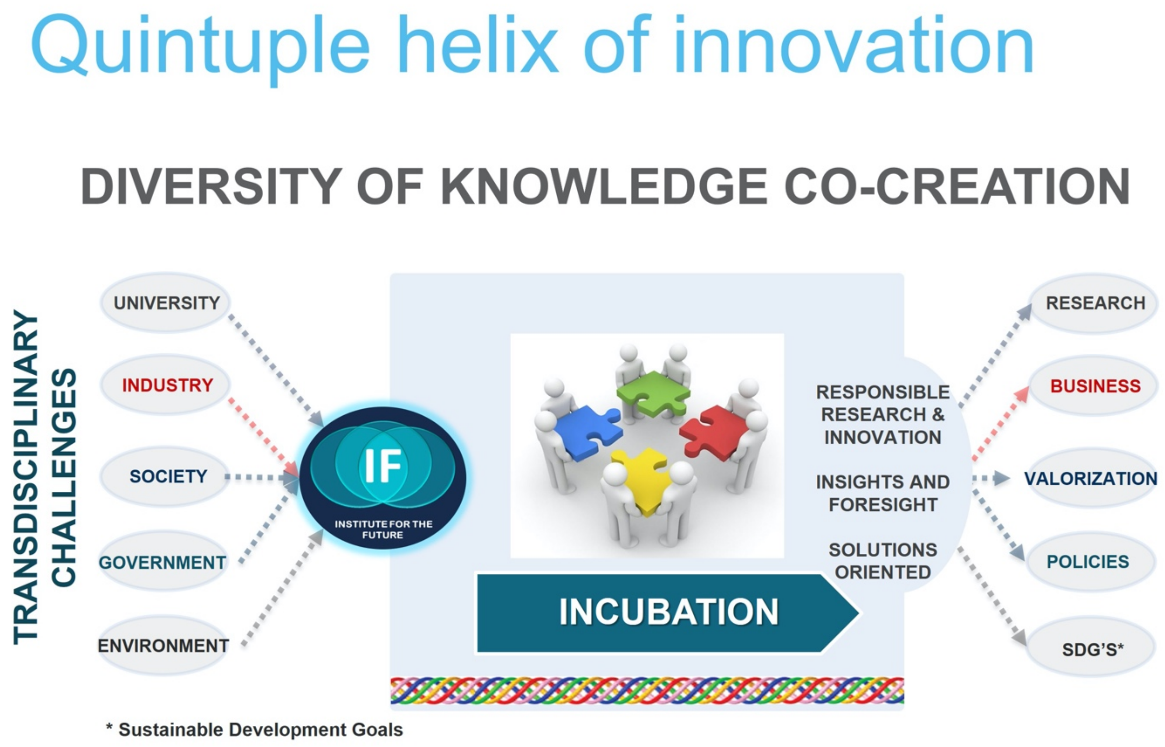

Figure 1. *The quintuple helix model of innovation the way it is applied at the Institute for the Future in Leuven.*

The academic board of the Institute launched the Transdisciplinary Insights Honours Programme[4] (HP-TDI) in 2016, in which Master's students from different faculties work in a transdisciplinary manner addressing complex challenges with the involvement of stakeholders. More recently, the master course was opened to Bachelor's students, Master's students and Ph.D. researchers, creating a space for co-creation and cross-fertilization among the participants and stakeholders, consolidating a transdisciplinary research incubator. As a result,

2 Carayannis, E.G.; Barth, T.D.; Campbell, D.F. The Quintuple Helix innovation model: Global warming as a challenge and driver for innovation. J. Innov. Entrep. 2012, 1, 2
3 https://en.wikipedia.org/wiki/Quadruple_and_quintuple_innovation_helix_framework
4 https://rega.kuleuven.be/tdi

several projects that initially started by teams at the HP-TDI, the initial incubation phase, have taken off as research projects. This HP-TDI was further developed by the academic board of the Institute over the years 2016 to 2019.

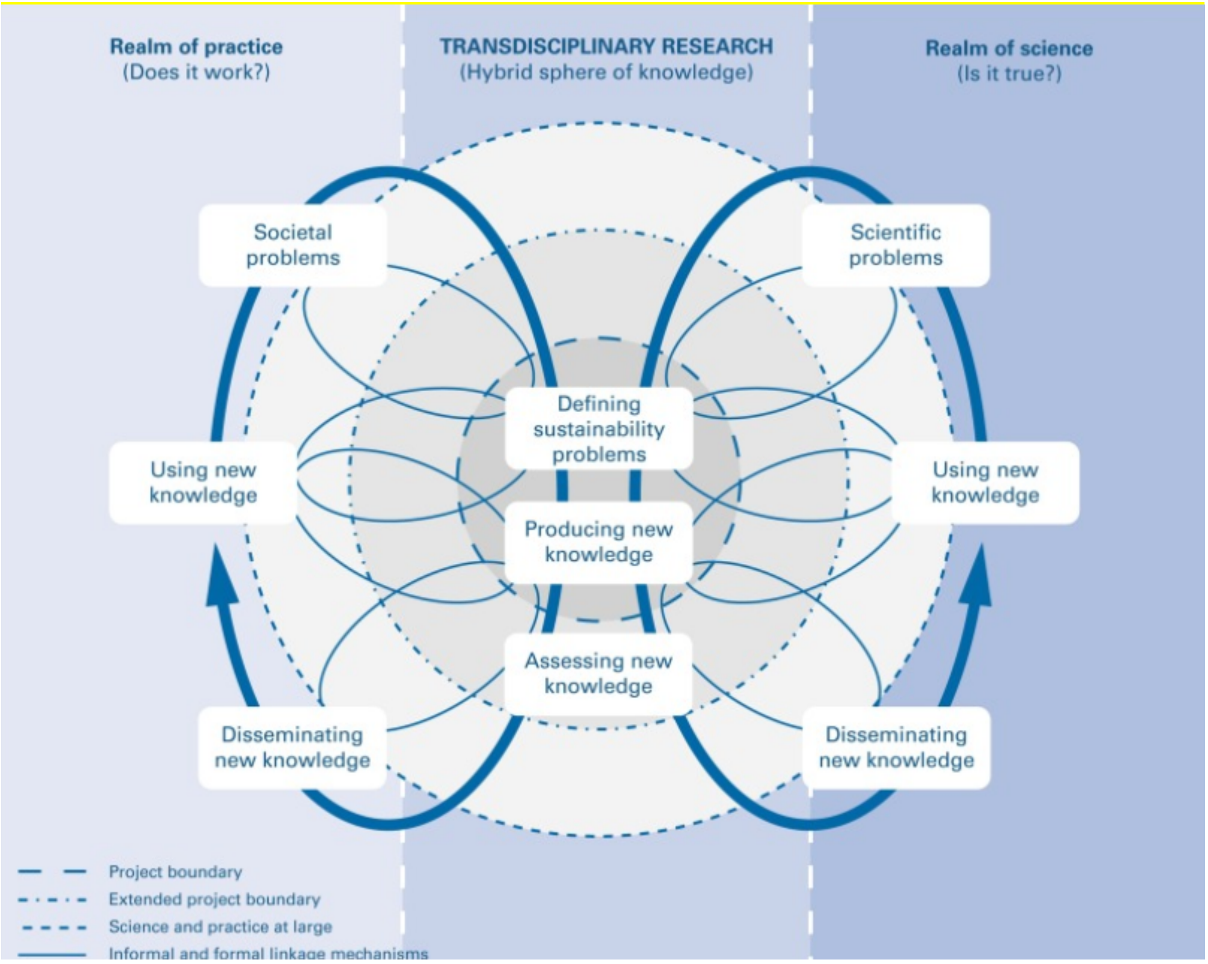

Figure 2. *How transdisciplinary research sits in the context of science and practice. From ten reflective steps* [5]

Through the contacts of the Leuven Institute for the Future with td-net Zürich[6]., HP-TDI initially relied heavily on the 10 reflective steps framework proposed by Christian Pohl[7] (Figure 2). Over the years, the practical experience at HP-TDI provided valuable elements for adapting this framework[8] resulting in the design of DF3, which stands for “Designing Feasible Futures Framework”. It was inspired not only by the ten reflective steps, but also by other frameworks such as “Grounded Action Design”[9] (Figure 3).

---

5 https://doi.org/10.1016/j.envsci.2019.08.011. Linking transdisciplinary research projects with science and practice at large: Introducing insights from knowledge utilization

6 https://transdisciplinarity.ch/en/uber-das-td-net/

7 https://transdisciplinarity.ch/en/transdisziplinaritat/forschungsprozess/; Pohl, C., Truffer, B. & Hirsch Hadorn, G. (2017). Addressing wicked problems through transdisciplinary research. In R. Frodeman, J. T. Klein & R. C. S. Pacheco (Eds.), The Oxford handbook of interdisciplinarity: Second Edition (pp. 319-331). Oxford, UK: Oxford University Press. https://doi.org/10.14512/gaia.26.1.10

8 https://doi.org/10.11116/TDI2017.1.3; https://doi.org/10.11116/TDI2020.4.2

9 https://doi.org/10.14512/gaia.28.4.3

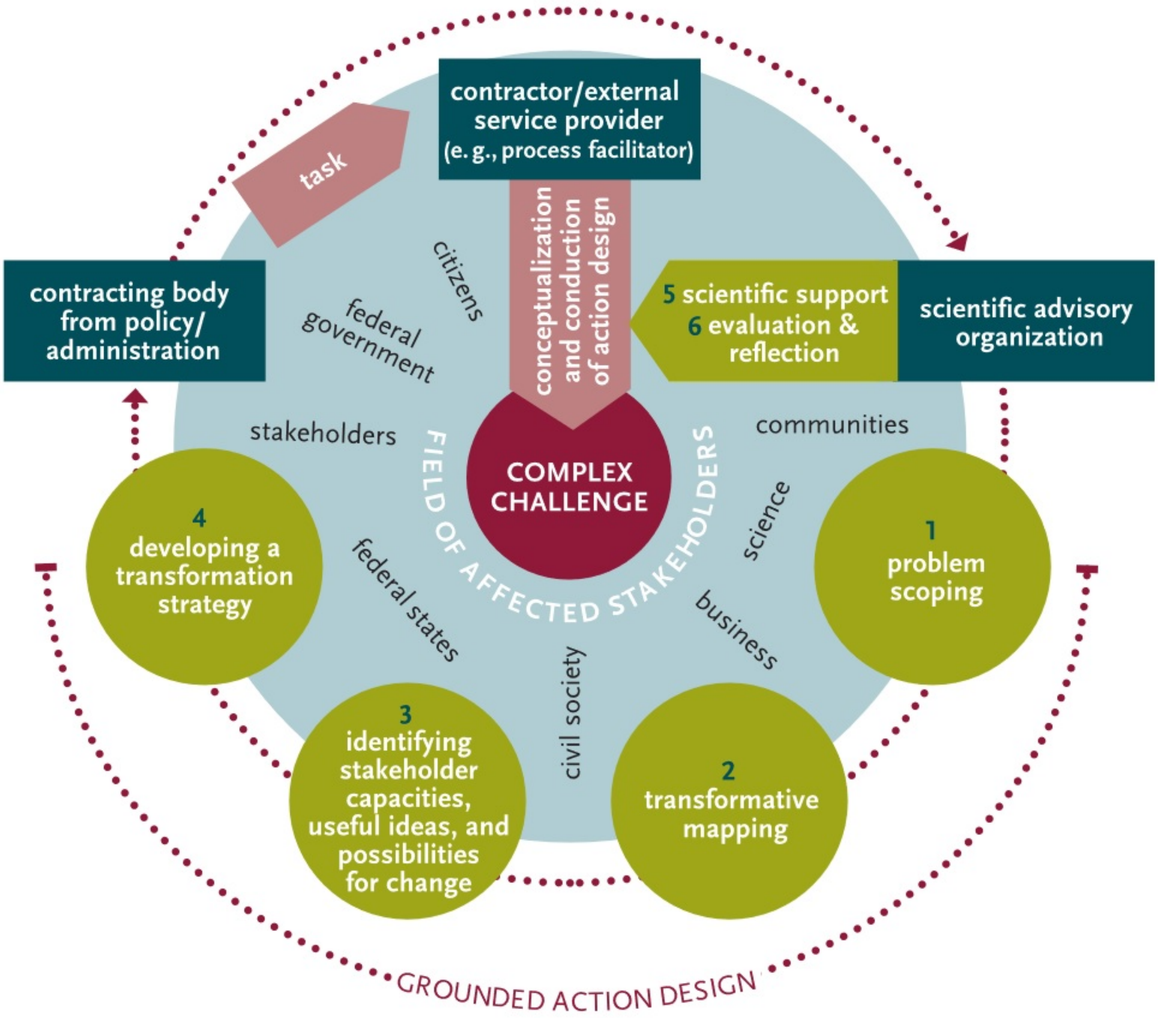

***Figure 3**. Building blocks and iterations as presented by the grounded action design framework*[10]

The crystallization of the framework was facilitated through a training by the European Panther programme in 2019, with the support of Flanders Business School[11]. The implementation of the DF3 framework was tested during the transdisciplinary work of the Coronavirus pandemic preparedness challenge[12] started early 2020 and was published as a methodological annex of the manuscript: Introducing Pandemic Preparedness Goals[13] in 2021.

The need for transdisciplinary frameworks

Wicked problems need to be addressed from multiple disciplines and need to integrate multiple knowledge systems (academic, experience, stakeholders). Such an integration is

---

[10] https://publications.iass-potsdam.de/rest/items/item_5009891_3/component/file_5009892/content
11 https://www.flandersbusinessschool.be/nl/opleidingen/european-panther-program
12 https://rega.kuleuven.be/if/pandemicpreparedness/home
13 Introducing Pandemic Preparedness goals. https://rega.kuleuven.be/if/pandemicpreparedness/introducing-pandemic-preparedness-goals

difficult, because of different cultures, habits and language, and different mental models of all the different parties involved. Misunderstandings and frictions can lead to toxic environments. Frameworks help to comprehensively integrate multiple disciplinary views and multiple bodies of knowledge. The framework in itself is a conceptual way to go through the steps that are needed to reach the goal, addressing the wicked problem. The framework is thus a reflection of the theory of change that the researchers have in mind, by which they assume they can make progress in addressing the wicked problem. It lies down the methodology that is planned to be used to integrate evidence and knowledge from multiple sources, to enhance the understanding of the wicked problem at hand, and to take steps for improvement.

Many frameworks for transdisciplinary research have been developed and published over the last years. GAIA Ecological perspectives for science and society compiled nine relevant frameworks published between 2017 to 2019[14]; each framework focuses on specific aspects either more oriented to principles or processes. How to chose a framework depends on how helpful the framework is for a particular wicked problem in a particular context[15]. An interesting framework that can inspire transdisciplinary work is theory U16. It is not necessarily a transdisciplinary framework, but it helps embody change, which is essential in transdisciplinary work

The need for the DF3 framework

Whatever the focus when addressing a wicked problem, all frameworks have some common and some complementary elements. The Institute for the Future identified five key elements to work with teams addressing a challenge: 1. Transdisciplinarity is not a linear but an iterative process, 2. Framing the problem is an essential step in problem identification and problem structuring, 3. System-based approach is necessary to understand the complexity of the topic, 4. Stakeholders are essential in co-creating knowledge from the beginning to the end, and 5. Transformation is a co-created emergent process. These five elements are conceptualized in the DF3 framework as four building blocks (or modules) in an iterative process.

We are continuing to develop the DF3 framework by designing associated methodologies for practical implementation in different socio-ecological circumstances. It is not that different from other frameworks, but **it places more emphasis on not getting stuck and moving forward.** Even if it feels that not enough attention has been paid to important aspects of the process, and that not enough knowledge has been gathered, the teams need to progress through the framework, improving in each next iteration. Systems are dynamic and it is impossible to deliver perfect solutions. Not moving forward will stall innovation and adaptation to new circumstances.

The DF3 framework outline

Therefore, the DF3 is an iterative transdisciplinary framework consisting of four modules: (1) problem framing, (2) complexity analysis, (3) multi-level stakeholder involvement, and (4)

---

14 https://www.oekom.de/_uploads_media/files/gaia_frameworks_for_td_research_110539.pdf
15 https://i2insights.org/2020/05/26/transdisciplinary-frameworks/
16 https://en.wikipedia.org/wiki/Theory_U, https://www.youtube.com/watch?v=WvNlfu4263Q; https://www.youtube.com/watch?v=byGUgoXFppE; https://www.youtube.com/watch?v=-9cv2hbYua4

designing possible futures (Figure 4). These four modules (framing, complexity, multi-level, and futures) are roughly sequentially worked through, but there is a lot of back and forth moving between exercises exploring the four blocks. Also iterations are not necessarily clearly delineated, the team may feel a new iteration has to be started even when the previous one has not gone through all modules. However, in general, an iteration process is completed once a team works together throughout the four modules. A new iteration can then start, building upon the insights resulting from the previous one. Between iterations, reflection is built in to take stock of the knowledge and process, and explicitly assess the learnings, which are then taken forward to the next iteration.

As a transdisciplinary team cycles through the building blocks of the DF3 framework, three types of knowledge are being generated in which 1. Systems knowledge is related to the knowledge of the current problem or situation *(it is about what is),* 2. Target knowledge is related to the desired futures and values *(it is about what should be)*, and 3. Transformation knowledge is related to the necessary steps to move from the current to the desired future *(It is about how to get from what is to what should be)*.[17], [18] The concept of the three types of knowledge was introduced initially in 1997 by ProClim (Forum for Climate and Global Change, Swiss Academy of Sciences) [19] and further developed over the last years as key core element of transdisciplinary research. [20] Figure 4 links the four building block in the context of the three types of knowledge.

---

[17] https://realkm.com/2021/02/17/three-types-of-knowledge/

[18] Hadorn, G., Biber-Klemm, S., Grossenbacher-Mansuy, W., & Hoffmann-Riem, H. (2008). *Handbook of Transdisciplinary Research* (1. Aufl. ed.). Dordrecht: Springer Netherlands. - Chapter 2
The Emergence of Transdisciplinarity as a Form of Research. Authors: Gertrude Hirsch Hadorn, Susette Biber-Klemm, Walter Grossenbacher-Mansuy, Holger Hoffmann-Riem, Dominique Joye,
Christian Pohl, UrsWiesmann and Elisabeth Zemp

[19] https://portal-cdn.scnat.ch/asset/7d12f07d-8a2c-56e5-9388-3dfc946cd9f8/1122?b=49f2591a-63d1-5adb-866c-2cefb4f34384&v=97d18713-8a31-5e4b-bea2-f1f63980ae97_0&s=Hio3RUQIM9SFOQcOUWotdzQ-W93Cw5xx4R5qvyWZcdtw3b4bzkaB56mDGz9joenHS8zdohZJG6rNLtY5DMwmMcks-cNlmiFOMTodiDJrZYhw81daEF2UTj_4Pdwob52_MfvZTAw_FT5edRpRofTg137krecUoeUgYs0VoZne3JI

[20] https://realkm.com/2021/02/17/three-types-of-knowledge/

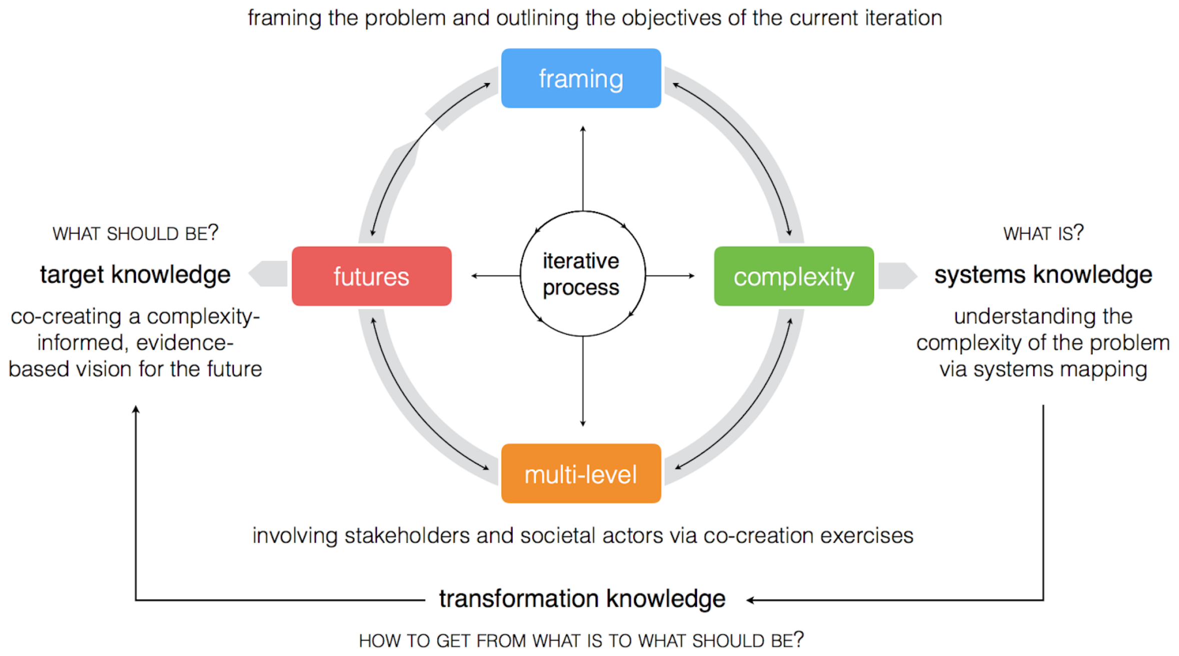

Figure 4. *The DF3 framework iterates along 4 modules (framing, complexity, multi-level and futures) generating 3 types of knowledge (systems, target and transformation). The learning teams are encouraged to act upon the transformation knowledge.*

The four modules of the DF3 framework:

1. **Framing**. The learning team frames the problem and outlines the objectives of the iteration with the support of an initial group of stakeholders.
2. **Complexity**. The learning team aims to deepen its understanding of the problem via systems mapping, causal loop diagrams and case studies to unravel patterns, relationships and interdependencies. This involves qualitative and quantitative analyses of the identified data sources, and putting them into a broader systems context. This results in systems knowledge.
3. **Multi-level**. The learning team identifies a broader set of stakeholders and societal actors via exercises (e.g. actor constellation exercises). The learning team subsequently involves the group of identified actors via co-creation workshops, focus group discussion and webinars, in order to expand its systems knowledge required for the challenge.
4. **Futures**. The learning team co-creates a complexity-informed, evidence-based vision for the future with the identified actors (target knowledge). Possible scenarios and roadmaps to achieve the desired targets are also drafted (transformation knowledge).

Essential concepts that need to be understood and addressed

When using any transdisciplinary framework, teams need to understand some essential concepts. These can be introduced through workshops or can be covered in different ways such as knowledge clips, books or literature. As much as possible these concepts should be introduced when they are relevant, and with visuals, such as little movies.

Wicked problems

Good short movies

https://www.youtube.com/watch?v=HrWbicvDLPw

https://www.youtube.com/watch?v=O8FMBBVb71k

https://www.youtube.com/watch?v=qUH5XOPF8pc

https://www.youtube.com/watch?v=HEQc83Xm8Hg

Primary characteristics of wicked problems

Rittel and Webber (1973)[21] identified ten primary characteristics of wicked problems:

1. There is no definitive formulation of a wicked problem, i.e. even the definition and scope of the problem is contested;
2. Wicked problems have no 'stopping rule', i.e. no definitive solution.
3. Solutions to wicked problems are not true-or-false, but good-or-bad in the eyes of stakeholders.
4. There is no immediate and no ultimate test of a solution to a wicked problem.
5. Every (attempted) solution to a wicked problem is a 'one-shot operation'; the results cannot be readily undone, and there is no opportunity to learn by trial-and-error.
6. Wicked problems do not have a clear set of potential solutions, nor is there a well-described set of permissible operations to be incorporated into the plan.
7. Every wicked problem is essentially unique.
8. Every wicked problem can be considered to be a symptom of another problem.
9. The existence of a discrepancy representing a wicked problem can be explained in numerous ways.
10. The planner has no 'right to be wrong', i.e. there is no public tolerance of initiatives or experiments that fail.

The role of values in wicked problems

Brian Head about wicked problems in public policy[22].

---

[21] Rittel, H W J and M M Webber (1973) 'Dilemmas in a General Theory of Planning', Policy Sciences, 4:2.

[22] Head, Brian. (2008). Wicked Problems in Public Policy. Public Policy. 3.

Figure 1: ‘Wicked’ as a combination of complexity, uncertainty and divergence

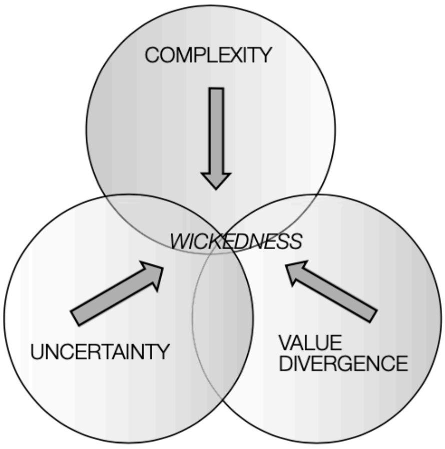

Figure 5. *Importance of values*

Wicked problem gridlocks:

1. *Approaching a wicked problem as a tame problem (aka trying to find technical solutions)*

Denying that a problem is wicked, or not understanding the value of transdisciplinarity, can lead to wicked problems being treated as tame, thinking that for any problem there is a technical solution (“technofix”). When you read this guide, you have probably already overcome this problem, you have already assessed that the challenge you are facing is a wicked problem. Tame problems have technical solutions, wicked problems need to be approached in a transdisciplinary way. Approaching a tame problem in a transdisciplinary way may lead to lots of frustration because of the slow progress of a transdisciplinary approach. Approaching a wicked problem with a technical solution will only increase the problem.

This is how De Fries et al[23] are describing it:

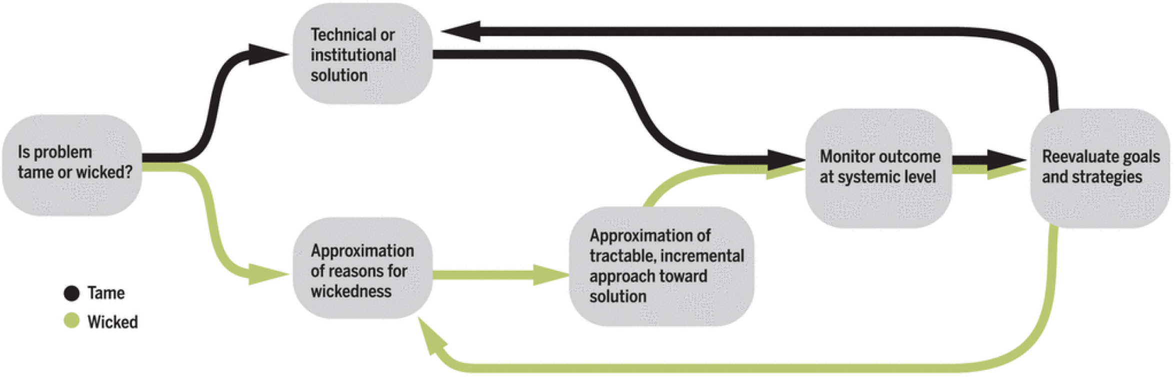

Fig. 1. Decision flowchart for wicked problems in ecosystem management. Such an approach can help to avoid trap A (falsely applying a technical, tame solution to a wicked problem) or trap B (inaction from overwhelming complexity).

Figure 6. *The trap of treating a wicked problem as a tame problem*

[23] DeFries et al, Science 21 April 2017

2. *Being discouraged to address a wicked problem because you don't fully understand it*

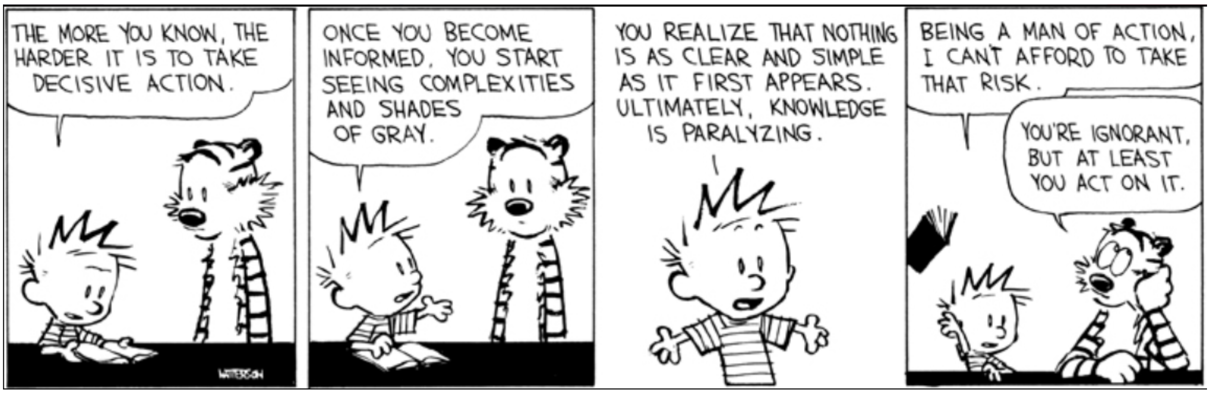

Figure 7. *The trap of paralysis*

"Think big, start small, act now, before everything becomes too late."

Barnabas Suebu, governor of the Indonesian province of Papua

Climate change has shown that being paralyzed by the complexity of a wicked problem can be detrimental in the long term. It is essential to take the first step in addressing a wicked problem as early as possible, because the problem unfolds itself while working on finding a way for improvement. That is why we need small iterative steps. While acting, you get a better understanding of the problem, the iterations are designed to each time act small (1 iteration) and move forward.

We are learning by doing: the way to start is … to start: try, fail, learn, improve, restart. This is old wisdom from the Persian philosopher Rumi (1207-1273 CE).

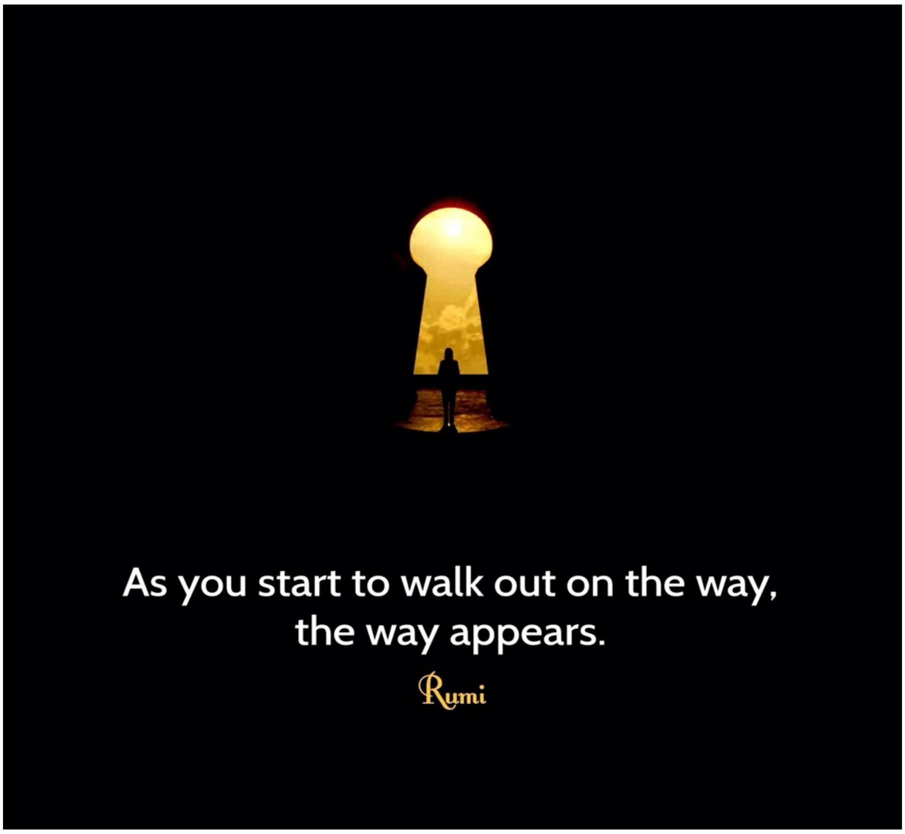

Figure 8. *Wisdom of the Persian philosopher Rumi (1207-1273 CE).*

Transdisciplinarity

IF's vision of transdisciplinarity, is reflected by the LERU definition (Figure 9).

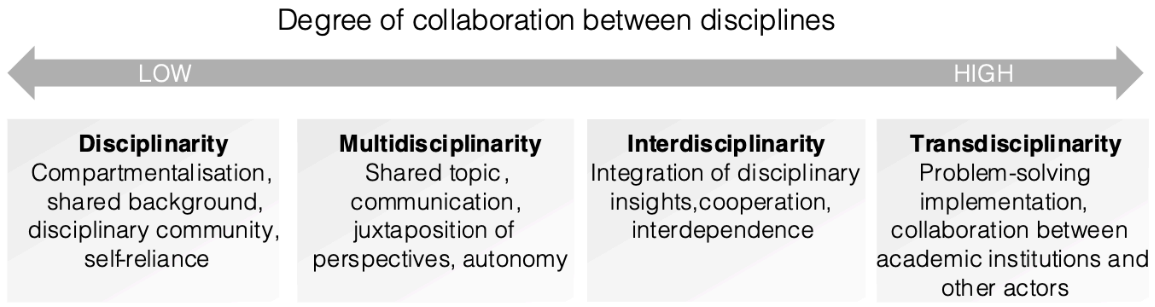

Figure 1. Key concepts for collaborative research between disciplines (interdisciplinarity). Inspired by Klein (2014).

Figure 9. *LERU statement of degrees of collaboration between disciplines. Taken from* [24]

Complexity

What is complexity, and what are the implications for addressing societal challenges? Through literature (e.g. Chapman[25]) and knowledge clips[26].
Systems thinking. Short movies explaining concepts[27].

Three types of knowledge

As the teams cycles through the modules of the DF3 framework, three types of knowledge are being generated[28] (Figure 10):

1. **Systems knowledge**. This is knowledge about *what is*. What does the current system (*e.g.* our society, the environment, our economy, *etc*.) look like? How does it work? What works well? What doesn't?
2. **Target knowledge**. This is knowledge about *what should be*. What should the system look like? What is our vision for the future? What are the desired goals and targets? What has to change?
3. **Transformation knowledge**. This is knowledge about *how to get from what is to what should be*. What knowledge do we need to transition from the current system to the desired system? What transformations of our medical, socioeconomic and political institutions are needed to reach the desired targets?

24https://www.leru.org/publications/interdisciplinarity-and-the-21st-century-research-intensive-university
25https://www.academia.edu/20173655/Complexity_and_Creative_Capacity_Rethinking_knowledge_transfer_adaptive_management_and_wicked_environmental_problems
26 Complexity Theory Overview
27 https://srinijanarthanam.medium.com/systems-thinking-simplified-dce12fc44220

28 https://transdisciplinarity.ch/de/transdisziplinaritat/was-ist-td/drei-arten-von-wissen/

Through transdisciplinary learning efforts, these three types of knowledge can contribute to societal impact by creating communication between and co-creation among science, politics and practice.

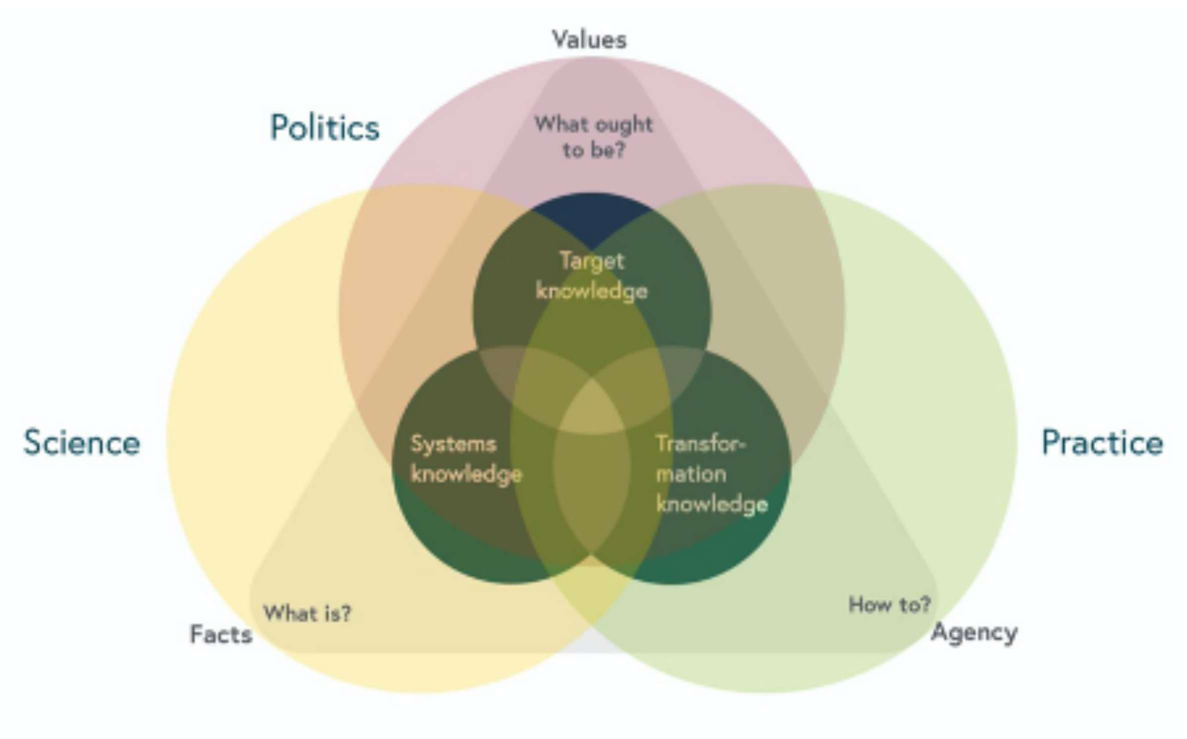

Figure 10. *Three types of knowledge and their positioning to achieve societal impact. From* [29]

29 https://transdisciplinarity.ch/de/transdisziplinaritat/was-ist-td/drei-arten-von-wissen/
