# Supplementary material for: Transdisciplinary training to address challenges in genomic epidemiology of infectious diseases
Source: Front Public Health. 2025 Dec 17;13:1713182. doi: 10.3389/fpubh.2025.1713182 (PMC12753939; doi:10.3389/fpubh.2025.1713182)
Supplement: Supplementary file 2 [file Data_Sheet_2.pdf]

VEME2022 Module 4: From Trees to Public Health Policy
Challenge case: How to judge on the need for border closing when a new SARS-CoV-2 variant of concern is reported.
"The case of the discovery of the Omicron variant during South Africa's surveillance efforts, and the decision to close borders with South Africa"

The COVID-19 pandemic has brought to the forefront the critical role of genomic sequencing within the context of pandemic response. As of July 2022, over 11 million SARS-CoV-2 sequences have been generated and shared by more than 190 countries around the world (GISAID). This abundance in sequencing data has allowed for an unprecedented level of precision[1] at which we can track the evolution of the SARS-CoV-2 virus, across the full spectrum of scales ranging from within-host[2] and transmission within a single hospital[3,4], to the municipal[5], national[6,7] and cross-national scales[8,9]. This is done within the framework of infectious disease genomic epidemiology, where genomes are used to reconstruct the spread of a given epidemic with the aid of phylogenetic trees that depict the shared ancestry of the pathogen[10].

Such analyses are increasingly more accessible due to the decrease in cost of genomic sequencing[11] coupled with advances in the speed and throughput of sequencing technologies[12], open and accessible protocols[13], and improvements in the usability of computational tools[13,14]. This decrease in barriers has allowed countries around the world to establish their own national genomic surveillance systems. Such national efforts not only help monitor the pandemic within the country, but also allow to track the evolution of the virus across national borders.

An exemplary case of cross-national collaboration in genomic surveillance can be seen in Africa, where Africa CDC's Pathogen Genomics Initiative has supported sequencing efforts across the continent, through the establishment of common sampling strategies, regional reference laboratories and operational protocols[15]. Because of this, researchers have been able to characterize the spread and progression of the SARS-CoV-2 pandemic in Africa, even in the presence of inconsistent case data. They showed that early during the pandemic, outbreaks in African countries were predominantly initiated by importations from Europe and followed by within-continent spread as the pandemic unfolded[9].

Most of these large scale genomic epidemiological studies are carried out by researchers at academic institutions. Nonetheless, as shown by the African Pathogen Genomics Initiative, partnerships between academics, public health practitioners and the broader policy sector must be in place in order to obtain the necessary amount of data to effectively track the pandemic. Beyond academic studies, monitoring and identifying emerging SARS-CoV-2 variants of concern or interest has been the most notable

application of genomic sequencing during the pandemic. Such variants refer to SARS-CoV-2 strains that contain a constellation of mutations that result in the virus being more transmissible, virulent or immune-evasive, which results in significant community transmission[16]. Up to date, a total of 13 VOCs and VOIs have been identified by the WHO, each with a corresponding Greek letter name. Because of their increase in transmissibility and risk of breakthrough infections, monitoring of such variants is of critical importance to public health decision makers.

However, policy makers and public health officials might not all have the necessary expertise in evolutionary virology and phylogenetics (which is often found in academic departments) required to accurately interpret genomic epidemiology analyses. Conversely, evolutionary virologists and molecular epidemiologists may not be fully aware of the concerns of policy makers when designing analysis plans and communicating about discoveries of new variants of concern.

With the goal of bridging this gap between researchers, public health practitioners and policy experts, WHO-PAHO organized a series of webinars on genomic epidemiology during the months of September and October of 2021. As preparation to present at this webinar, organizers from this VEME 2022 module set up a meeting with a diverse group of stakeholders in order to better understand the current challenges in the use of genomic epidemiology for decision making. The meeting was approached with a transdisciplinary lens, with participants across multiple levels of the genomic surveillance-to-policy pipeline, including academics and experts in molecular diagnostics and sequencing, public health, genomic epidemiology, and policy. The following ten questions summarize common sentiments shared during the meeting:

1. *How big is the gap between scientists and decision makers?*
2. *How can we bridge gaps early on to devise epidemiological & phylogenetic studies that are both feasible scientifically and useful for policy guidance?*
3. *Why would governments invest in sequencing beyond surveillance efforts?*
4. *How useful are genomic studies for decision makers in absence of a good integration of epidemiological data?*
5. *How useful are retrospective genomic epidemiology studies for decision makers?*
6. *Which limitations do decision makers experience in genomic epidemiology studies?*
7. *What does a sustainable genomic epidemiology system geared to inform decision makers look like?*
8. *Can missing or inaccurate data associated with sequences derail decision making?*
9. *What is the added value for decision makers of sequencing a large number of genomes?*
10. *How do we turn trees into transmission control measures?*

Thus, the VEME 2022 module From Trees to Public Health Policy was designed as a way to identify and challenge some of the tensions underlying these ten questions. We aim to do this by bringing together researchers, practitioners and decision makers under a transdisciplinary framework developed at the KU Leuven's Institute of the Future[17]. We will introduce participants to essential concepts of this framework by using the problem of COVID-19 travel restrictions as a case study.

Genomic epidemiology has played a crucial role in instilling travel bans to countries where variants of concern or interest have been detected. The most notable example is the travel ban on incoming flights from South Africa after the country's identification of the Omicron variant[18]. Despite South Africa's leading efforts in genomic surveillance, the country has been the target of unwarranted bans punishing their openness and transparency in science. In addition, travel restrictions were shown to have come after the Omicron variant had already spread to Belgium and multiple other countries[19,20]. While it is questionable whether these travel bans had any significant effect on slowing down the spread of the Omicron variant, such travel bans risk to lead to less scientific transparency by disincentivizing countries to share information and potentially exacerbating political tensions. The stigma associated with these measures has also dire economic consequences, as seen by the state of South Africa's tourism industry[21]. However, governments across the world instilled these bans despite the fact that scientific studies had already shown how limited their effect was (3 to 5 days delay), while other measures were proven much more effective[22].

While public health scientists focus on the scientific rigor of public health measures, policy makers must consider the political dimension of the problem. Here, because of the delayed pandemic response of many governments during the early stage of the pandemic, the lived experiences of politicians incentivized them to take such "precautionary measures". Moreover, national economic interests may lead governments to opt for outwardly facing measures instead of instilling policies that may disrupt the national economy. Once we consider these factors, we can appreciate that responding to emerging variants with travel bans and other decisions informed by genomic epidemiology, presents us with a complex problem with no easy solution, where science, politics and economic factors are often in opposing tension. Therefore, we believe that it is essential for a transdisciplinary team to come together and identify existing knowledge gaps, and brainstorm and co-create strategies that account for the complexity of the problem. We hope this VEME 2022 module serves as the starting point to identify better and more inclusive ways in which genomic epidemiology can better support public health policy and practice.

References

1. Ladner, J. T., Grubaugh, N. D., Pybus, O. G. & Andersen, K. G. Precision epidemiology for infectious disease control. *Nat. Med.* **25**, 206 (2019).
2. Lythgoe, K. A. *et al.* SARS-CoV-2 within-host diversity and transmission. *Science* **372**, (2021).
3. Stirrup, O. *et al.* Evaluating the effectiveness of rapid SARS-CoV-2 genome sequencing in supporting infection control teams: the COG-UK hospital-onset COVID-19 infection study. *medRxiv* 2022.02.10.22270799 (2022) doi:10.1101/2022.02.10.22270799.
4. El Moussaoui, M. *et al.* Evaluation of Screening Program and Phylogenetic Analysis of SARS-CoV-2 Infections among Hospital Healthcare Workers in Liège, Belgium. *Viruses* **14**, 1302 (2022).
5. Bollen, N. *et al.* Exploiting genomic surveillance to map the spatio-temporal dispersal of SARS-CoV-2 spike mutations in Belgium across 2020. *Sci. Rep.* **11**, 1–8 (2021).
6. Butera, Y. *et al.* Genomic sequencing of SARS-CoV-2 in Rwanda reveals the importance of incoming travelers on lineage diversity. *Nat. Commun.* **12**, 1–11 (2021).
7. Candido, D. S. *et al.* Evolution and epidemic spread of SARS-CoV-2 in Brazil. *Science* **369**, 1255–1260 (2020).
8. Lemey, P. *et al.* Untangling introductions and persistence in COVID-19 resurgence in Europe. *Nature* **595**, 713–717 (2021).
9. Wilkinson, E. *et al.* A year of genomic surveillance reveals how the SARS-CoV-2 pandemic unfolded in Africa. *Science* **374**, 423–431 (2021).
10. Grubaugh, N. D. *et al.* Tracking virus outbreaks in the twenty-first century. *Nature Microbiology* **4**, 10–19 (2018).
11. Pennisi, E. A $100 genome? New DNA sequencers could be a 'game changer' for biology, medicine. https://www.science.org/content/article/100-genome-new-dna-sequencers-could-be-game-changer-biology-medicine.
12. Gardy, J., Loman, N. J. & Rambaut, A. Real-time digital pathogen surveillance — the time is now. *Genome Biol.* **16**, 1–3 (2015).
13. Artic Network. https://artic.network/ncov-2019.
14. Hadfield, J. *et al.* Nextstrain: real-time tracking of pathogen evolution. *Bioinformatics* **34**, 4121–4123 (2018).
15. Ngbonga, T. Interim operational guidance on SARS-CoV-2 genomic surveillance in Africa: An updated guide. *Africa CDC* https://africacdc.org/download/interim-operational-guidance-on-sars-cov-2-genomic-surveillance-in-africa-an-updated-guide/ (2021).
16. Tracking SARS-CoV-2 variants. https://www.who.int/activities/tracking-SARS-CoV-2-variants.
17. Coronavirus Pandemic Preparedness Project. *KU Leuven Institute for the Future* https://rega.kuleuven.be/if/pandemicpreparedness/home; https://rega.kuleuven.be/if/pandemicpreparedness/introducing-pandemic-preparedness-goals; (2021).
18. Marcus, L. & Neild, B. Travel restrictions by country following the Omicron variant

outbreak. *CNN* https://www.cnn.com/travel/article/coronavirus-omicron-variant-travel-restrictions/index.html (2021).
19. Martuscelli, C. & Cokelaere, H. New coronavirus variant detected in Belgium as Europe bans Southern Africa travel. *POLITICO* https://www.politico.eu/article/von-der-leyen-commission-to-propose-southern-africa-travel-shutdown-over-new-coronavirus-variant/ (2021).
20. Mendelson, M. *et al.* The political theatre of the UK's travel ban on South Africa. *Lancet* **398**, 2211 (2021).
21. Kapela, N. South Africa tourism was just starting to rebound. Then omicron arrived. *The Washington Post* (2022).
22. Chinazzi, M. *et al.* The effect of travel restrictions on the spread of the 2019 novel coronavirus (COVID-19) outbreak. *Science* **368**, 395–400 (2020).
