# Supplementary material for: Transdisciplinary training to address challenges in genomic epidemiology of infectious diseases
Source: Front Public Health. 2025 Dec 17;13:1713182. doi: 10.3389/fpubh.2025.1713182 (PMC12753939; doi:10.3389/fpubh.2025.1713182)
Supplement: Supplementary file 3 [file Data_Sheet_3.pdf]

Supplementary Figures

P	E	S	T	E	L	H
Government policy	Economic growth	Population growth rate	Technology incentives	Weather	Discrimination laws	Feeling well
Political stability	Exchange rates	Age distribution	Level of innovation	Climate	Antitrust laws	Disease markers
Corruption	Interest rates	Career attitudes	Automation	Environmental policies	Employment laws	Hospital capacity
Foreign trade policy	Inflation rates	Safety emphasis	R&D activity	Climate change	Consumer protection laws	Expertise of health care personnel
Tax policy	Disposable income	Health consciousness	Technological change	Pressures from NGO's	Copyright and patent laws	Effectiveness of vaccines
Labour law	Unemployment rates	Lifestyle attitudes	Technological awareness		Health and safety laws	Drug resistance
Trade restrictions		Cultural barriers				

Supplementary Figure S1: PESTEL + H analysis framework. This table provides examples of factors corresponding to each of the different categories: Political, Economic, Social, Technological, Environmental, Legal, and Health (PESTEL+H). Each column represents a category within the framework, and the rows list examples of factors that fall under the corresponding category.

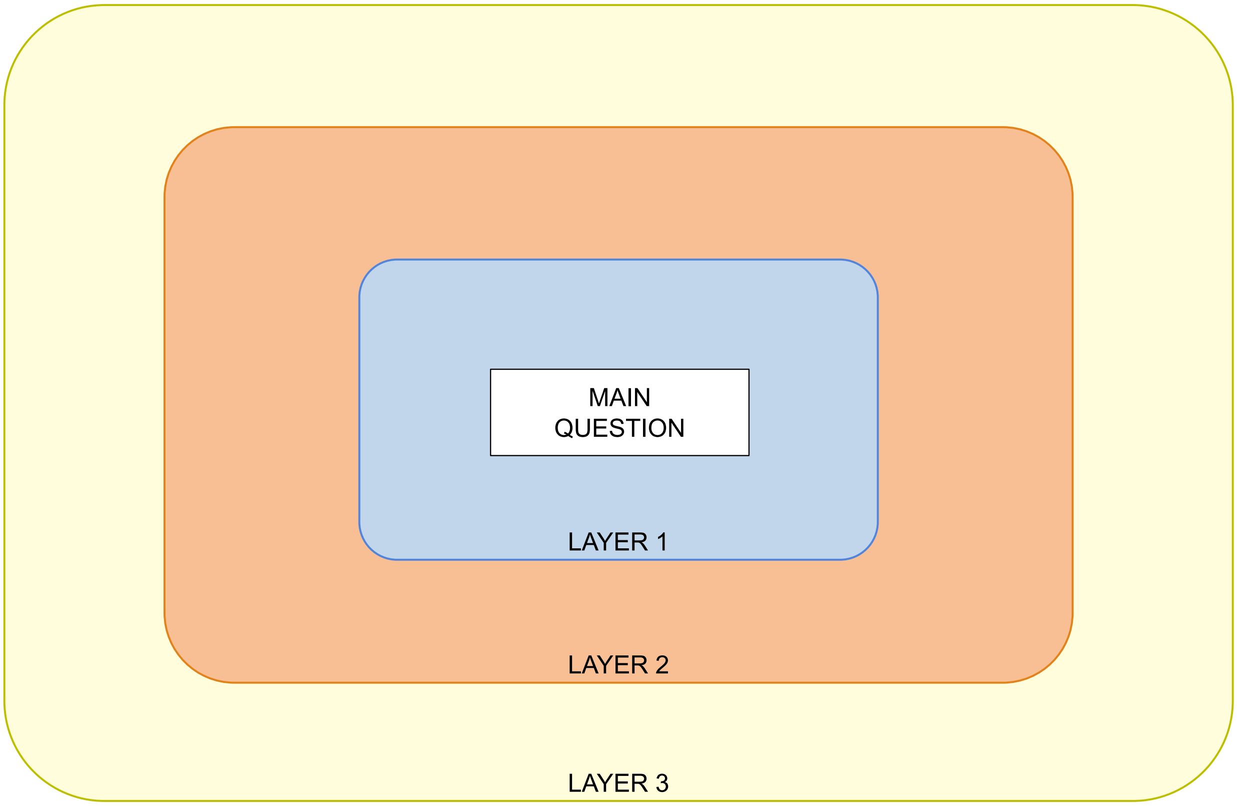

Supplementary Figure S2: Actor constellation exercise for Multi-Level Stakeholder Analysis. The diagram illustrates a method for visualizing stakeholder relationships across different levels of influence, with the central focus of the analysis represented as the "Main Question" at the core. Layer 1 encompasses stakeholders with the most direct influence or involvement in the main question requiring high level involvement, while Layer 2 includes those with indirect influence, often by influencing actors in Layer 1. Layer 3 comprises stakeholders with a broader or more distant connection to the main question, who may still be impacted by the outcomes or exert influence on the other layers. The different levels in the actor map roughly represent different levels of involvement in the “multi-level” stakeholder involvement approach

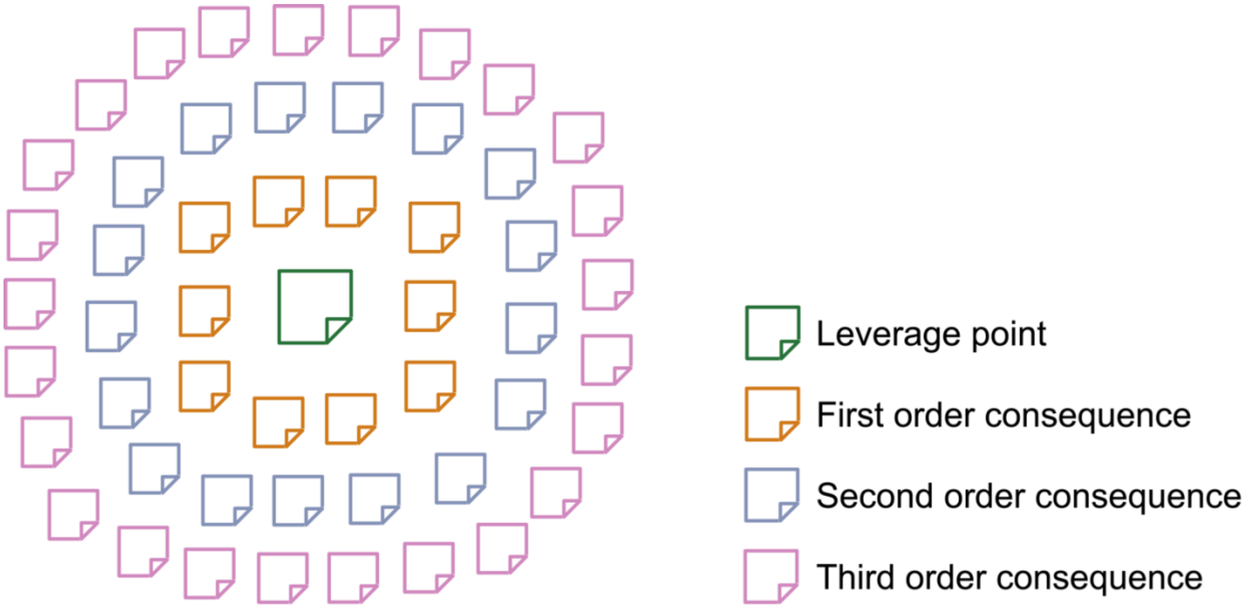

Supplementary Figure S3: Futures wheel exercise. The diagram depicts a Future Wheels exercise, a method used to explore the potential downstream consequences of an intervention. The central "Leverage Point" represents the intervention itself, while the surrounding layers of sticky notes illustrate the cascading effects, categorized by order of consequence (direct, second-order, third-order). Each sticky note symbolizes a specific consequence, with arrows indicating causal links between them, demonstrating how initial actions can ripple outwards and trigger further, often unforeseen, consequences.
