# Supplementary material for: Transdisciplinary training to address challenges in genomic epidemiology of infectious diseases
Source: Front Public Health. 2025 Dec 17;13:1713182. doi: 10.3389/fpubh.2025.1713182 (PMC12753939; doi:10.3389/fpubh.2025.1713182)
Supplement: Supplementary file 4 [file Data_Sheet_4.pdf]

Evaluation of VEME Conference Module 4: From Trees to Public Policy
Draft 11/23/2022
Peter M Rabinowitz MD MPH

<u>Outline:</u>

1. Executive summary
2. Description of module process and evaluation approach
3. Observations notes during steps in process
4. Lecture evaluations
5. Comments from Reflection Sessions
6. Additional Feedback Sessions with participants
7. Pre and Post survey- analysis of results
8. Other evaluation data

Executive Summary:

The following Is an evaluation of a four-day workshop module during the 2022 VEME conference that focused on transdisciplinary problem solving related to genomic information. Participants in the workshop worked in groups to develop "Theory of Change" approaches to a "wicked problem" of their choosing, with the end result being a completed Theory of Change and accompanying materials.

Evaluation methods included administration of a pre-and post-test questionnaire, daily group self-evaluation of happiness and frustration levels during workshop stages, and confidential written feedback at the end of the workshop.

The workshop was attended by professionals with diverse backgrounds including public health background, genetics/bioinformatics, policy, public government affairs, mathematics, and virology.

Results of the pre and post-test survey indicated that participants reported significantly increased familiarity with the concept of a "wicked problem", increased confidence in their ability to use systems thinking, to work in interdisciplinary teams, and to engage in stakeholders to develop solutions to such problems. Respondents also increased their agreement with the statement that transdisciplinary approaches are useful and effective.

Description of Workshop processes and steps

The workshop was conducted over a 4-day period as one module of a larger conference on Viral Evolution and Molecular Epidemiology (26th annual VEME conference). Participants that had selected this module went through the following steps during the 4-day module.

Day 1:

Introduction, Baseline Questionnaire:

Day 1 started with some introductory lectures about wicked problems and the logistics of creating a Theory of Change (TOC) to address them. Participants had already been provided with a summary of a wicked problem, using as a case example the controversy over the reporting of the discovery of the Omicron variant of SARS Co-V-2 by South Africa and the resultant travel and trade restrictions imposed on that country. At this point, participants completed a baseline evaluation questionnaire regarding interdisciplinary problem solving.

Groups and Safe Space

The group was divided into two subgroups of 10-11 people, and since many participants were limited English speaking (Spanish primary language), one group was primary Spanish speaking with headsets for simultaneous translation. There was an icebreaker that consisted of sharing information about favorite dishes as well as geographic locations using post it notes and a map. Following this, each group then went through a safe space exercise to discuss ground rules for group discussions. This involved individuals writing out requests for safety in discussions on post it notes, then comparing to others and assembling a composite list of desired qualities for discussion including mutual respect, honoring privacy, inclusiveness and empathy. After a second round to ensure all aspects were being captured, the groups signed an written agreement about honoring these safe space attributes.

Theory of Change Introduction

Facilitators went over the Theory of Change process, and emphasized that the groups could use the Omicron case study that had been provided or choose another problem.

Individual Theory of Change development

Each individual was asked to write out a theory of change for a particular problem (Omicron or other), with the sections of why, how, what (actions) and what (outcomes).

Sharing of individual Theories of Change

Participants then shared their individual TOCs with the rest of the group.

Refinement of the “Why”

The groups then worked to come to a consensus about a group TOC, getting as far as determining the “why” and a bit of the “how” but not getting all the way through the TOC process. This involved discussion of things like whether to focus on biomedical vs. societal issues.

Explanatory lecture

Merging back into the larger conference, participants listened to a plenary talk about interdisciplinary problem solving focused on pandemic preparedness.

Further development of TOC:
The day ended with a final session working on the "How" and "what" of the TOC.

Day 2:

Reflection:
Before advancing, each group took time to reflect on the first day's activities, and to make graphs of levels of group happiness and frustration and how they varied during the day. (see below). The two groups then shared these graphs with each other.
The two groups also shared the draft TOCs they had worked on with each other, outlining the general focus of their wicked problem.

Introduction to systems mapping:
There was a general lecture outlining the concept of systems mapping and providing examples. This lecture introduced the group to the PESTEL-H checklist (

Further discussion of key question (why)
The groups did an exercise of refining the question to focus on for the Theory of Change, putting several choices up on a board and then individuals using dots to select the specific choice.

PESTEL Exercise
Having refined the question, the group then did an exercise to identify factors using the PESTEL-H framework,

Voting on Factors:
The groups then used dots and sticky notes to select the most important PESTEL-H factors to include in the systems map.

Systems mapping:
The group then worked together to make a systems map that incorporated the selected PESTEL-H factors. The system map included arrows or loops to designate relationships between the concepts.

Day 3:

Reflection:
As in Day 2, Day 3 began with a group session to graph frustration and happiness from the previous day.

Introduction to use of systems maps and actors
There was another talk about the use of systems maps, including the need to choose a leverage point to focus on for the theory of change

Choosing leverage points:
The groups then discussed choosing a leverage point in their systems maps. One way this was done was to find the factors/concept with the most connecting arrows.

Revisiting key question
The discussion of leverage point led to a discussion of a refined question (Why) for each group's theory of change.

Introduction to Actors
There was a lecture about the importance and different categories of actors involved in our relevant systems, including which actors had more power and influence than others. Terms such as dormant, demystifying, and discretionary were discussed.

Voting on actors:
Each group worked to identify different categories of actors, (Power, Urgency, Legitimacy, etc.) and vote on their category and importance and where they fit into the systems map.

Refinement of Systems Map
Groups further refined their systems map to include actors and leverage points as well as PESTEL-H factors, essentially creating 3 layers of the map. Interactions between actors were included in the current map. As the groups refined the map, it was an opportunity to continue to refine the central question as well and the scale of the change (local, national, regional, global, etc.) It was also an opportunity to refine the "how" of the theory of change, as well as the "what" (actions).

Day 4:

Reflection:
Once again, groups began the day with a review of the previous day's events and a graphing of happiness and frustration during the day.

Introduction to Futures:
This introductory lecture covered the concept of futures planning and the creation of a futures wheel to anticipate first, second, and third order consequences of the proposed change in the TOC.

Futures Wheel:
Each group worked on creating a futures wheel with their key intervention in the center, and radiations of 1st, 2nd, and 3rd order consequences, with indicators for whether the consequences would be positive or negative. This put additional focus on the "what" aspects.

Actions:
With the experience of the futures wheel, the groups were able to finish refining the "what" aspects of the theory of change.

Sharing of TOCs.
The two groups then had the opportunity to fully present their theories of change to the other groups

Final Reflections:
The group rated the levels of happiness and frustration during this final day by creating one more set of graphs. Participants were also given an opportunity to provide comments about the overall workshop, both positive and negative. There was also completion of a final evaluation questionnaire.

Evaluation Methods:

1. Pre and Post Test Evaluation Survey:

Participants completed a pre-test and a post-test evaluation form that included five questions regarding confidence and familiarity regarding the content of the workshop. The form asked participants to indicate degree of agreement by placing an X on a line from labeled on the left "strongly disagree" and on the right "strongly agree".
These questions are shown in Box 1:

1. "I am familiar with the concept of a "wicked problem"
2. "I am confident in my ability to use "systems thinking" to address complex/wicked problems"
3. "I am confident in my ability to form and work in "transdisciplinary teams" to solve complex/wicked problems
4. "I am comfortable engaging with stakeholders about genomic information issues"
5. "Transdisciplinary processes are a useful and effective way to solve wicked problems":

Box 1: Pre and Post Test Questions

Responses on each form were converted to a 10-point scale. Mean scores for each question were calculated, as well as the change in that mean between the two timepoints, and a two-sided T-test used to calculate the level of statistical significance for the change in mean score.

2. Pyramid Response Regarding Decision Making

In addition to the 5 evaluation questions above, participants were asked, in both the pre and post test, to give an opinion about who should be primary in complex policy decision making about genomic issues, by indicating with a dot in the following pyramid.

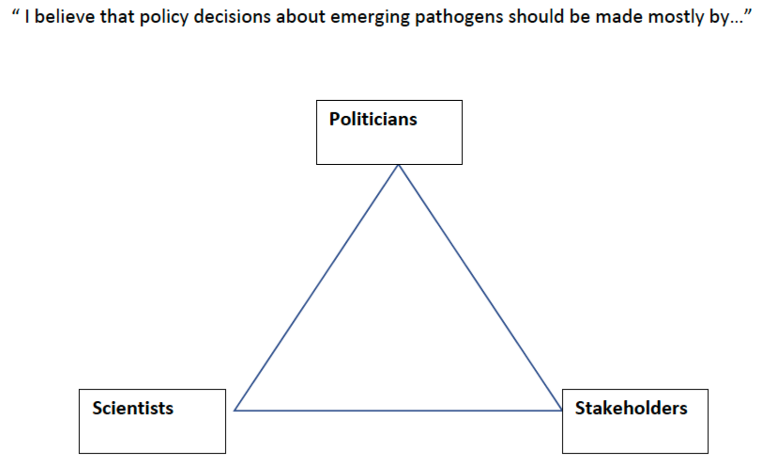

Figure 1: pyramid for pre and post-test response about decision-making about genomic issues.

Responses were scored categorically according to whether the dot was closest to any of the points of the triangle or to the center. A 2X4 frequency table was created for the results, and Chi-square statistic calculated to test the statistical significance of the difference in responses between the pre and post-test. Descriptive analysis assessed the pre-post changes in particular responses.

3. <u>Happiness and Frustration Graphs</u>

On a daily basis, each of the two groups completed a "happiness and frustration" graph for the previous day, specifying how the group's collective level of happiness or frustration fluctuated during different activities. These graphs were completed by a designated member of each group drawing on the graph while receiving direction from the rest of the group about what levels of happiness or frustration were being experienced at each timepoint and stage of the workshop.

4. <u>Additional Feedback Comments:</u>

At the final session of the workshop, participants were asked to anonymously provide 3 types of written feedback via writing comments on post-it notes. The 3 types of feedback were: 1) positive aspects of the course 2) negative aspects of the course, and 3) suggestions for improvement. These were classified into categories and are presented in their entirety.

Evaluation Results

<u>Background of participants</u>

In the pre-test survey, participants were asked to indicate as many professional backgrounds as they wished. 15 individuals reported having a public health background, 7 reported a genetics/bioinformatics background, 3 a policy maker background, and one each specifying

public government affairs, mathematician, and virologist. For the post-test survey, 11 participants reported a public health/medicine background, 7 genetics/bioinformatics, 3 policy makers, and one each microbiology and ID modeler.

Pre and Post Test Evaluation Survey:

Table 1 shows the results of participant responses to the questions in the pre and post-test evaluation survey. By the end of the workshop, participants reported increased familiarity with the concept of a "wicked problem", increased confidence in their ability to use systems thinking, to work in interdisciplinary teams, and to engage in stakeholders to develop solutions to such problems. Respondents also increased their agreement with the statement that transdisciplinary approaches are useful and effective. All of these increases were statistically significant.

**Question**	**Pre (N=21)**	**Post (N=17)**	**Change**	**P value**
**1. "I am familiar with the concept of a "wicked problem**	**5.31**	**9.15**	**3.84**	**9.3 E -08**
**1. I am confident in my ability to use "systems thinking" to address complex/wicked problems"**	**5.1**	**8.5**	**3.4**	**9.5 E -07**
**2. I am confident in my ability to form and work in "transdisciplinary teams" to solve complex/wicked problems**	**6.55**	**8.15**	**1.95**	**0.00068**
**3. I am comfortable engaging with stakeholders about genomic information issues"**	**6.26**	**8.44**	**2.18**	**0.001**
**4. "Transdisciplinary processes are a useful and effective way to solve wicked problems":**	**7.52**	**9.0**	**1.48**	**0.0042**

Table 1: Results of pre- and post-test question responses

Table 2 shows the responses to the "Pyramid question" regarding which groups should be primary in decision-making about the use of genomic information. Between the pre-test and the post-test, there was a trend toward a greater proportion of respondents choosing "center" (indicating shared decision making) rather than a particular group. The proportion choosing the center increased from 10/20 (50%) to 14/17 (82%), while the proportions choosing either scientists, politicians, or stakeholders decreased, although none of these trends achieved statistical significance (chi square 0.73, p=0.8).

	**SCIENTISTS**	**POLITICIANS**	**STAKEHOLD**	**CENTER**	**TOTAL**
**BEFORE**	5	3	2	10	**20**
**AFTER**	1	1	1	14	**17**
**TOTAL**	**6**	**4**	**3**	**24**	**37**

Table 2: Responses to Pyramid Question about Decision Making.

Happiness and Frustration Graphs

Separate graphs were completed for the two groups (Figures 2A and 2B). The two graphs tell a slightly different story, indicating some differences in experience between the two groups. Group 1 reported some episodes of frustration on days 1-3, including during the initial development of a group Theory of Change (day 1), the systems mapping (day 2), and the voting on significant actors (stakeholders) (day 3). By day 4 however, levels of frustration were low and happiness high.
Group 2 reported high levels of frustration and declining happiness on day 1, but much less frustration on day 2, and by day 4 the group reported low levels of frustration and high levels of happiness similar to Group 1.

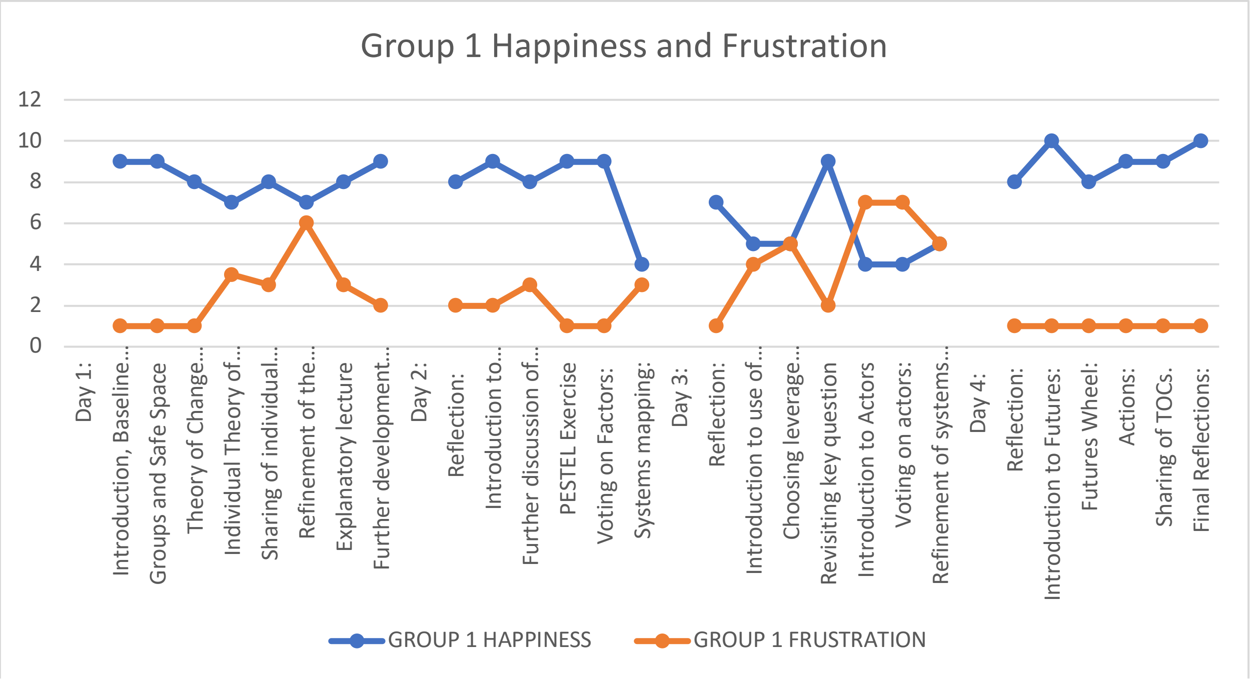

Figure 2A: Group 1 Happiness and Frustration over the course of the workshop

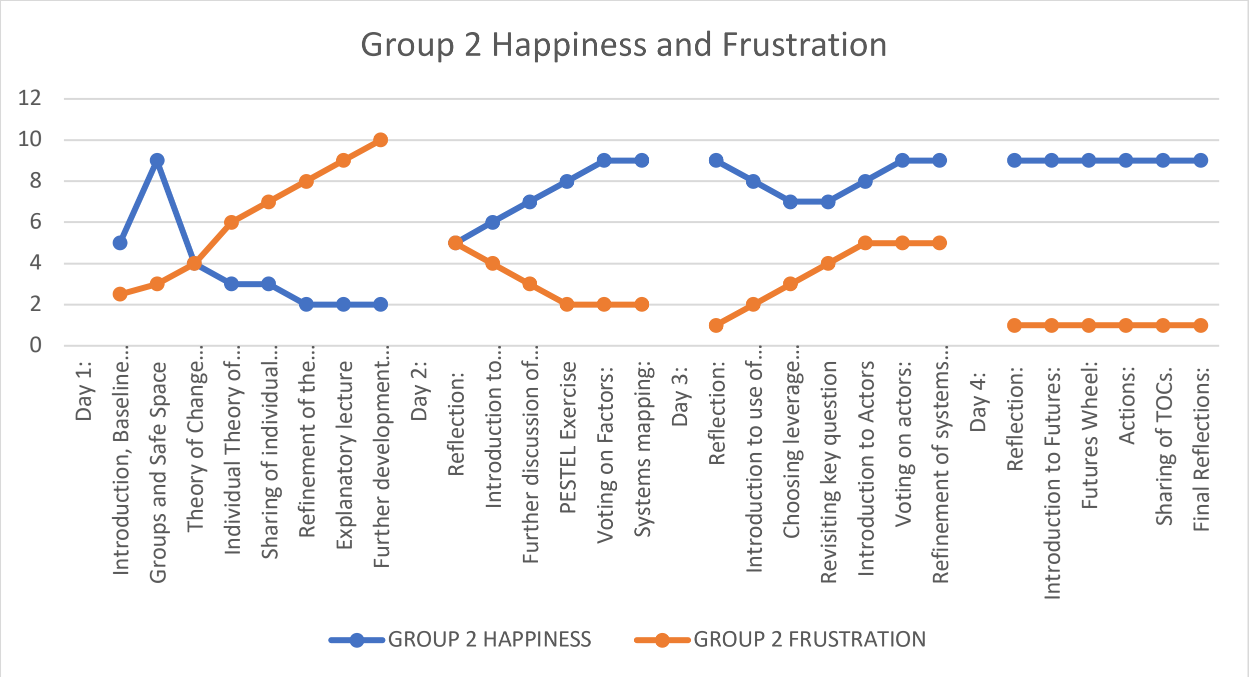

Figure 2B: Group 1 Happiness and Frustration over the course of the workshop

<u>Additional Feedback Comments:</u>

The positive and negative comments are listed below, as well as the suggestions for improvement. Key positive aspects noted by participants included the overall course structure, the innovative methodology, the instructors/facilitators, and the lectures. Negative aspects noted included the fact that some felt that the methods were confusing, especially at first, and that the workshop at times felt rushed. Suggestions for improvement included greater pre-workshop preparation with materials for self-study, changes in the overall length of the course and time for individual exercises, exercises focused on group dynamics, organized group dinners, and greater connection to bioinformatics issues.

Positive comments

<u>Overall course structure</u>

- Milestones defined
- The theory/practice went really well
- (Good) to have transdisciplinary experience
- It was nice to have a lot of variation in the group and an opportunity to meet with people of different backgrounds. Definitely something that is a great and enriching experience
- Excellent application of transdisciplinary theory to (a) real world wicked problem

- Transformative workshop
- Put in practice the transdisciplinary approach in solution of a wicked problem
- I think it's an excellent course (for) the first time
- The product we got is extremely good

Methodology

- Team building was expertly accomplished
- I really like the methodology of the training and different topics
- Interactive engaging methodology
- methodology used
- a lot of opportunity for learning by doing
- interactive discussions
- good way of starting and closing the workshop together
- dynamic way of learning
- group engagement
- good methodology
- novelty
- The methodology used to conduct the work
- The active and engaging methodology

Instructors

- Instructors/facilitators were knowledgeable
- good instructors
- The facilitators

Lectures

- great lectures!
- clear lectures

Specific components

- Power of connections made at personal level with the group cannot be underestimated
- Safe space - good exercise!
- Food - good icebreaker

Critiques

Overall approach

- The beginning is very confusing. It is important that the instructions are clearer at the beginning. Also, everything is fast.
- Uncertainty
- Use is(in) more cases?

- It's not clear how the methodology could be applied in real life (with decision makers, for example)
- Without sense of direction, choices made by group felt often rudderless and prone to drift from one exercise to another.

<u>Time constraints and intensity</u>

- Time constraint (distribution of the time for the activities)
- Little time
- Insufficient time for any activities.
- Duration of the module (time)
- Pace too intense at times

Suggestions for improvement

<u>Pre-course preparation:</u>

- Course preparation materials- Previous reading material can help.
- Sharing the background literature, a bit earlier would help to get more familiar with the theory in advance and prepare for the workshop
- Having readings beforehand should have been encouraged
- Share the information with several days prior to the workshop
- Have the reading material at least one week in advance
- Make the sent document more useful
- Improved communication in advance for better preparation
- A bit more timely information about the content before the course

<u>Ideas for exercises/activities</u>

- Possible exercise/training about group dynamics/communicating in a timely efficient manner
- possible exercise to challenge your own bias exercise
- suggest prior to the workshop that they put videos on YouTube of the methodology
- Organized dinner/drinks/breaks for people in the module (to learn about each other better)
- More talks about policy-makers and their perspective (similar to ana bento)
- I liked the exercises and teambuilding, but really wanted a better sense of outline and direction. Perhaps demo of how (this) approach works to solve a toy problem. Would have helped on day 1.
- The systems map can be more connected with bioinformatics

<u>Timing:</u>

- possibly extend the time to allow for more talks/thoughts
- Perhaps it could be a 5-day module since the half day modules are very focused on other modules. It would take away perhaps some of the time pressure
- Maybe better working time

- Free day in the middle instead of Friday off
- By the end it was easier to work together as we got a sense of timing and got to know each other better. Maybe it is possible to have this happen before the 4th day, i.e., to speed up this process (maybe more moderation?)
- Need more time - add additional day

Other suggestions:

- Elaborate this module in Spanish to expand its application
- Change module name
